# Faithful or evasive? An empirical study on translation norm preferences of Chinese and American LLMs in Chinese official political and policy discourse

**DOI:** 10.3389/frai.2026.1869972

**Published:** 2026-08-10

**Authors:** Xinyu Zhu, Yu Yin

**Affiliations:** School of Foreign Languages, Southeast University, Nanjing, China

**Keywords:** Chinese official political and policy discourse, cross-cultural comparison, forced-choice translation ranking task, large language models, translation norm orientations

## Abstract

**Introduction:**

Large language models now handle a share of cross-lingual political translation, but no study has directly measured whether they follow stable normative preferences when doing so.

**Methods:**

We target Chinese-to-English translation of Chinese political and policy terms embedded in authentic official discourse. Mapping 52 publications across translation theory, LLM empirics, and AI alignment yields a five-dimensional Translation Norm Orientations (TNO) framework: Faithfulness (FN), Fluency (FLN), Cultural Adaptation (CAN), Ethical Hedging (EHN), and Foreignization Retention (FRN). Eight mainstream Chinese and American LLMs complete a Forced-Choice Translation Ranking Task (FCTRT) on 20 ideologically sensitive political terms, ranked across three repeated trials. Kendall’s W assesses ranking stability, Mann–Whitney U tests with BH-FDR correction test between-group differences, and hierarchical clustering identifies latent normative prototypes.

**Results:**

All eight models rank FN first; CAN, EHN, and FRN settle into a shared low-priority band beneath it. None of the five dimensions shows a statistically significant CN–US split. FN alone carries a medium effect size (Cliff’s *δ* = 0.50), which points to a difference in how strongly, not which way, the two groups lean. Cluster membership cuts across national lines rather than following it: four transnational profiles emerge, separated by foreignization tolerance rather than developer geography.

**Discussion:**

These results tie translation norm theory to AI evaluation and supply a benchmark other researchers can reuse to examine LLM-mediated political communication.

## 1 Introduction

Translation is not a transparent conversion of codes. In descriptive translation studies, translation norms constitute the central framework for analyzing how translators negotiate the tension between adequacy and acceptability, a negotiation that mirrors deep-seated power structures within specific sociocultural contexts (Toury, 1995). When the translating agent shifts from culturally situated humans to data-driven large language models (LLMs), this foundational premise faces an ontological rupture: the translator becomes a probabilistic system parameterized by billions of weights, and traditional investigative tools such as motivational inquiry and ethnographic interviews lose their epistemic footing.

The practical stakes of this shift are considerable. Leading LLMs already rival professional translators across general language pairs (Hendy et al., 2023; Jiao et al., 2023) and are penetrating vertical domains including journalism and government affairs (Hu and Li, 2023). A fundamental question thus arises: do these models exhibit stable, observable ranking tendencies when translating politically sensitive texts?

Converging evidence supports this concern. Ma et al. (2026) reveal that alignment strategies implicitly encode value hierarchies, and political discourse translation constitutes a cross-linguistic arena of power contestation where norm selection bears a structural relationship to cultural hegemony (Baker, 2018a; Venuti, 2017). When these latent tendencies intervene in the rendering of ideologically charged political texts, the sheer volume of translations driven by convergent alignment regimes stands to reshape the ecology of cross-linguistic political communication at an unprecedented scale.

The comparative dimension sharpens this concern. Wen and Tian (2024) have documented ChatGPT’s systematic semantic dilution of discourse bearing distinctly Chinese characteristics, and Yu and Liu (2024) observe that AI is actively reconfiguring cross-linguistic discourse power distributions. Yet whether Chinese and American models tend to preserve or attenuate ideological tension in translation remains empirically uncharted. Most studies confine themselves to qualitative single-model analyses or abstract value measurements detached from concrete translation scenarios (Sühr et al., 2025; Wang and Xie, 2024), and *post-hoc* evaluations of spontaneous outputs cannot isolate the causal contribution of specific normative orientations to translation decisions.

Accordingly, this exploratory study targets Chinese-to-English translation of Chinese political and policy terms in official discourse contexts, proposing Translation Norm Orientations (TNO) as the core construct and a Forced-Choice Translation Ranking Task (FCTRT) as the measurement instrument. Three questions are addressed:

*RQ1:* Do Chinese and American LLMs exhibit stable, observable ranking tendencies in translating Chinese political and policy terms, and how are these tendencies distributed across the five TNO dimensions?

*RQ2:* Are there statistically significant between-group differences in ranking preferences, and do such differences concentrate on specific dimensions or specific sensitive terms?

*RQ3:* Do normative preference clusters align with CN–US geopolitical boundaries, or are they driven primarily by training data and alignment strategies, revealing transnational descriptive model profiles?

## 2 Literature review and theoretical framework

### 2.1 Theoretical foundations of translation norm research

Since Toury (1995) established translation norms as an empirical category within descriptive translation studies (DTS), the field has bifurcated along two complementary axes. The descriptive–professional axis, advanced by Chesterman’s (1993) distinction between expectancy norms and professional norms (Chesterman, 2016), foregrounds how normative knowledge is transmitted and internalized by practicing translators. The critical–ethical axis, anchored in Venuti’s (2017) theorization of domestication and foreignization as strategies enacting cultural power, was extended by Berman’s (1999) advocacy for preserving source-text “otherness” through literal translation and complemented by Nord’s (2014) functionalist attention to contextual purpose. Baker (2018b) bridged the two traditions by pioneering a corpus-based empirical framework for translation universals, laying methodological groundwork for the computational approaches that followed. Crucially, all these frameworks presuppose a human agent capable of normative internalization through culturally embedded social interaction (Chesterman, 2016)—a premise that collapses when LLMs displace human translators.

### 2.2 Translation norm tendencies of large language models

Beyond matching human-level accuracy (Hendy et al., 2023; Jiao et al., 2023), leading LLMs display systematic normative tendencies in translation. Jiang et al. (2023) showed through corpus comparison that ChatGPT outputs exhibit pronounced simplification and explicitation—patterns highly isomorphic with “normalization” yet driven by statistical encoding of high-frequency parallel-corpus features rather than by semantic comprehension. Karpinska and Iyyer (2023) further observed recurrent “semantic bleaching” of culturally loaded terms in LLM literary translation. Value-laden biases compound these tendencies: the cultural orientations of translation outputs closely mirror pre-training data structures (Naous and Xu, 2025); ChatGPT tends to default to mainstream American cultural stances on sensitive topics (Torrielli, 2023); and ideological leanings can be objectively surfaced through specific lexical choices (Chen et al., 2024). For Chinese-to-English translation specifically, Xu and Tian documented systematic semantic dilution and smoothing when ChatGPT processes distinctively Chinese political discourse (Wen and Tian, 2024). What remains absent is direct measurement of models’ internal normative hierarchies under controlled conditions.

### 2.3 Cross-cultural measurement of AI value preferences

Beyond translation studies proper, computational social science has begun to probe the value preferences underlying LLM behavior. Zhong et al. (2024) found that a single model’s Hofstede cultural-dimension scores shift markedly with the language of the prompt, exposing high situational dependency in model value representation. Ma et al.’s (2026) cross-cultural evaluation further showed that Chinese and American models do not diverge along clean geopolitical lines; clustering is driven more by alignment strategy than by developer nationality—a finding that reframes the terms of cross-national comparison. Methodologically, Adilazuarda et al. (2024) and Sühr et al. (2025) caution that elicited value profiles are confounded by training-corpus homogeneity and that extending human scales to AI without measurement invariance risks an “ontological error.” Millière (2025) underscored that RLHF fosters only “shallow alignment” devoid of genuine normative internalization, so claims about AI “preferences” demand stricter evidential standards.

### 2.4 Research gaps and study positioning

Three gaps emerge from the foregoing review. First, AI translation metrics anchored to correctness (BLEU, MQM (Freitag et al., 2022)) cannot distinguish normatively convergent from faithfully equivalent translations. Second, value-preference measurement has not descended to concrete translation decisions, leaving a substantial ecological-validity deficit. Third, no cross-national framework isolates normative orientation from low-level linguistic competence (Hu and Li, 2023; Wang, 2021); systematic comparisons using ideologically sensitive Chinese political discourse remain virtually nonexistent. This study addresses these gaps through the TNO-based FCTRT.

### 2.5 Systematic literature mapping and TNO construction

To anchor the framework in disciplinary evidence rather than researcher presupposition, 52 publications were assembled into an analytical corpus organized across four strands: (A) canonical translation-theory works by Toury, Chesterman, Venuti, Baker, Nord, and Berman; (B) empirical studies on LLM translation behavior; (C) political discourse translation research; and (D) AI safety and alignment literature, notably Ouyang et al. (2022), Millière (2025), and Dahlgren Lindström et al. (2025).

**Figure 1:**
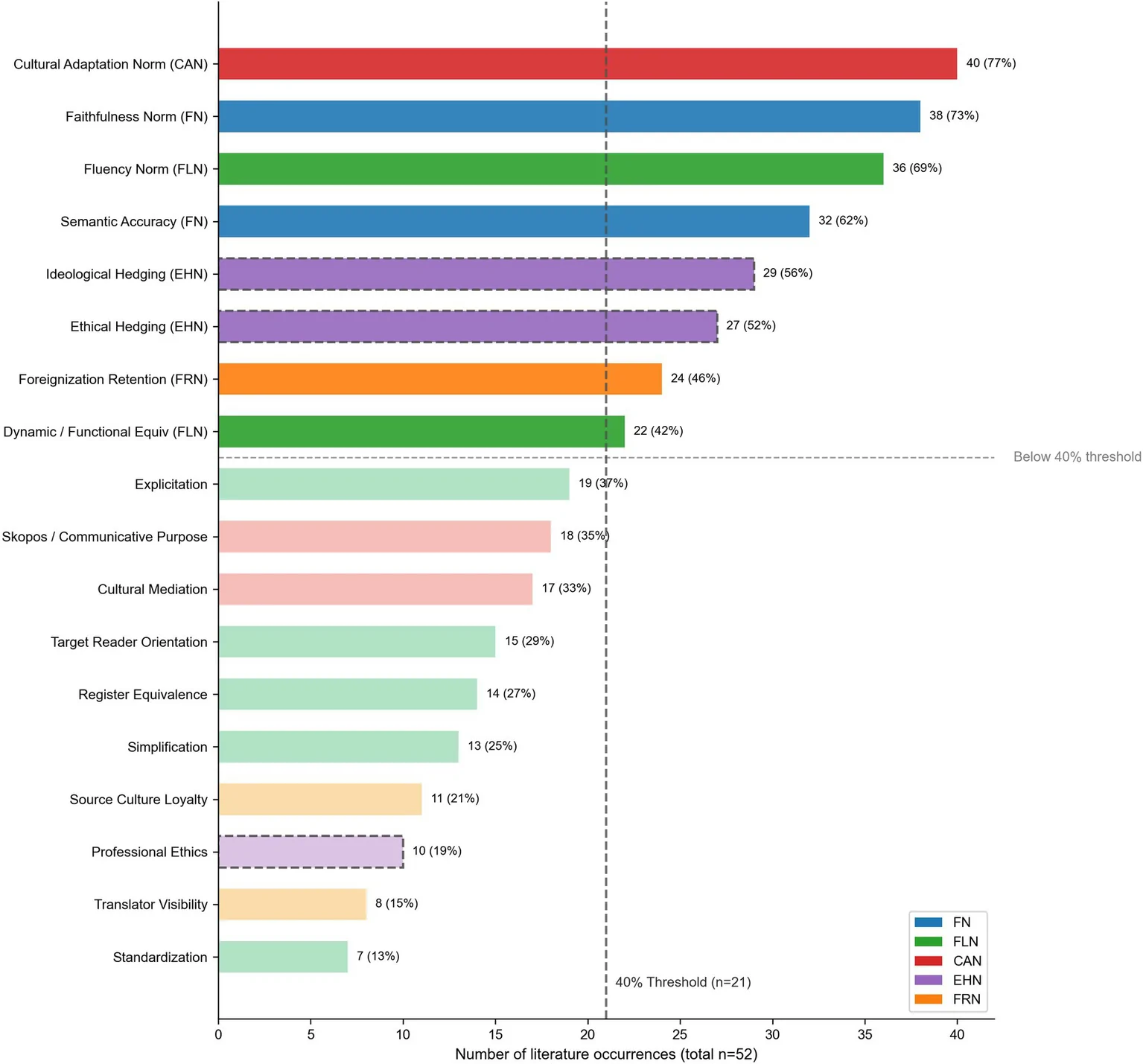
Overall frequency distribution of translation norm concepts in the literature (*n* = 52).

Two independent coders (Master’s-level Translation Studies) binary-coded all 18 candidate normative concepts across the corpus (1 = explicitly discussed, 0 = absent or marginal), working blind to each other’s judgments. Inter-rater reliability reached Cohen’s *κ* = 0.83 (Cohen, 1960), meeting the “substantial agreement” threshold (κ ≥ 0.61) (Landis and Koch, 1977; see Figure 1).

**Figure 2:**
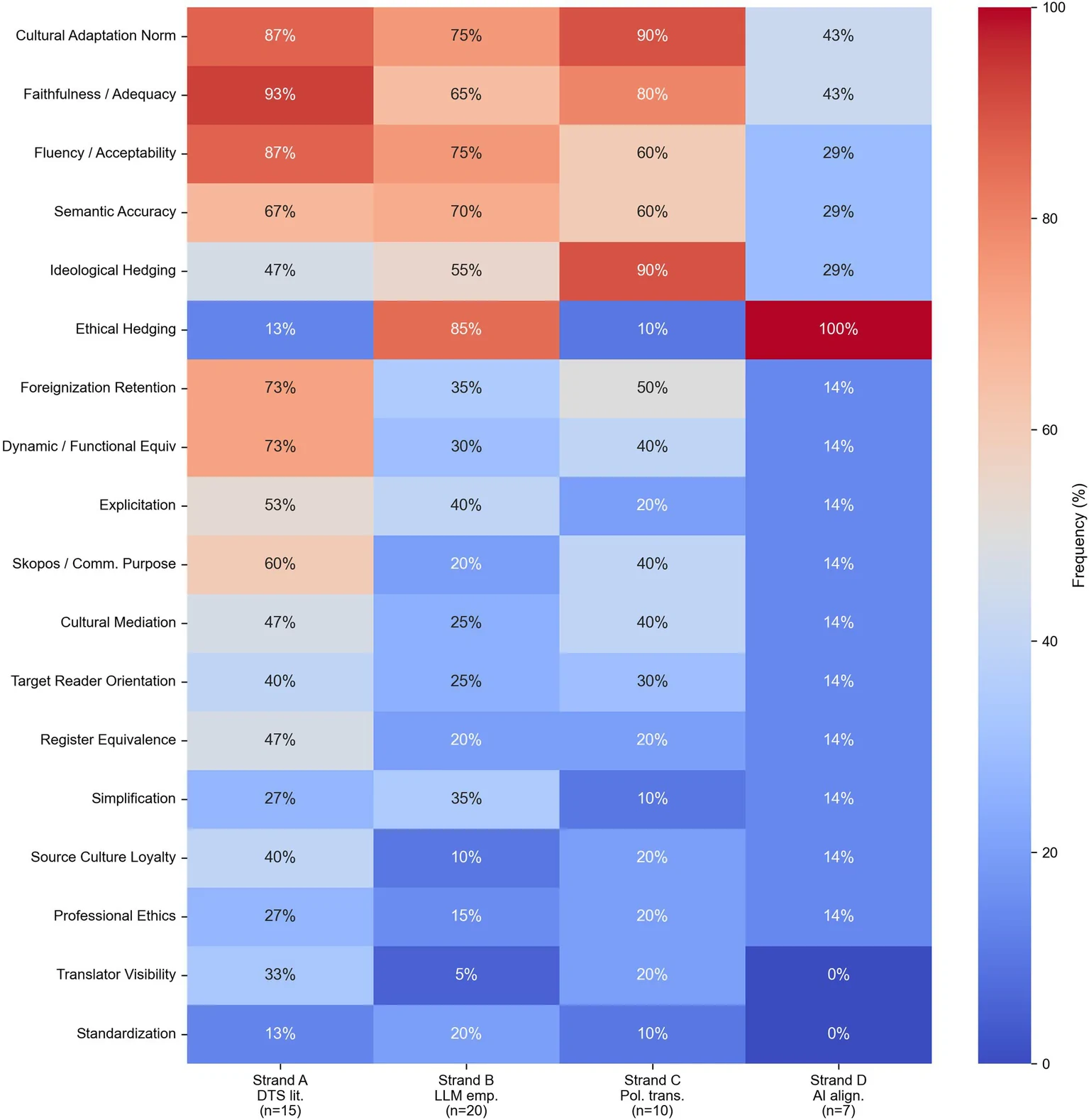
Matrix of occurrence frequency of 18 translation norm concepts in four literature sub-libraries.

The frequency data reveal three patterns: eight concepts exceeded the 40% threshold, entering the TNO candidate pool; low-frequency concepts were excluded; and Ethical Hedging (EHN) exhibited a sharply heterogeneous distribution—13% in traditional DTS yet 85–100% in AI/alignment literature—furnishing objective grounds for treating EHN as an AI-specific dimension (see Figure 2).

### 2.6 The TNO five-dimensional framework

**Figure 3:**
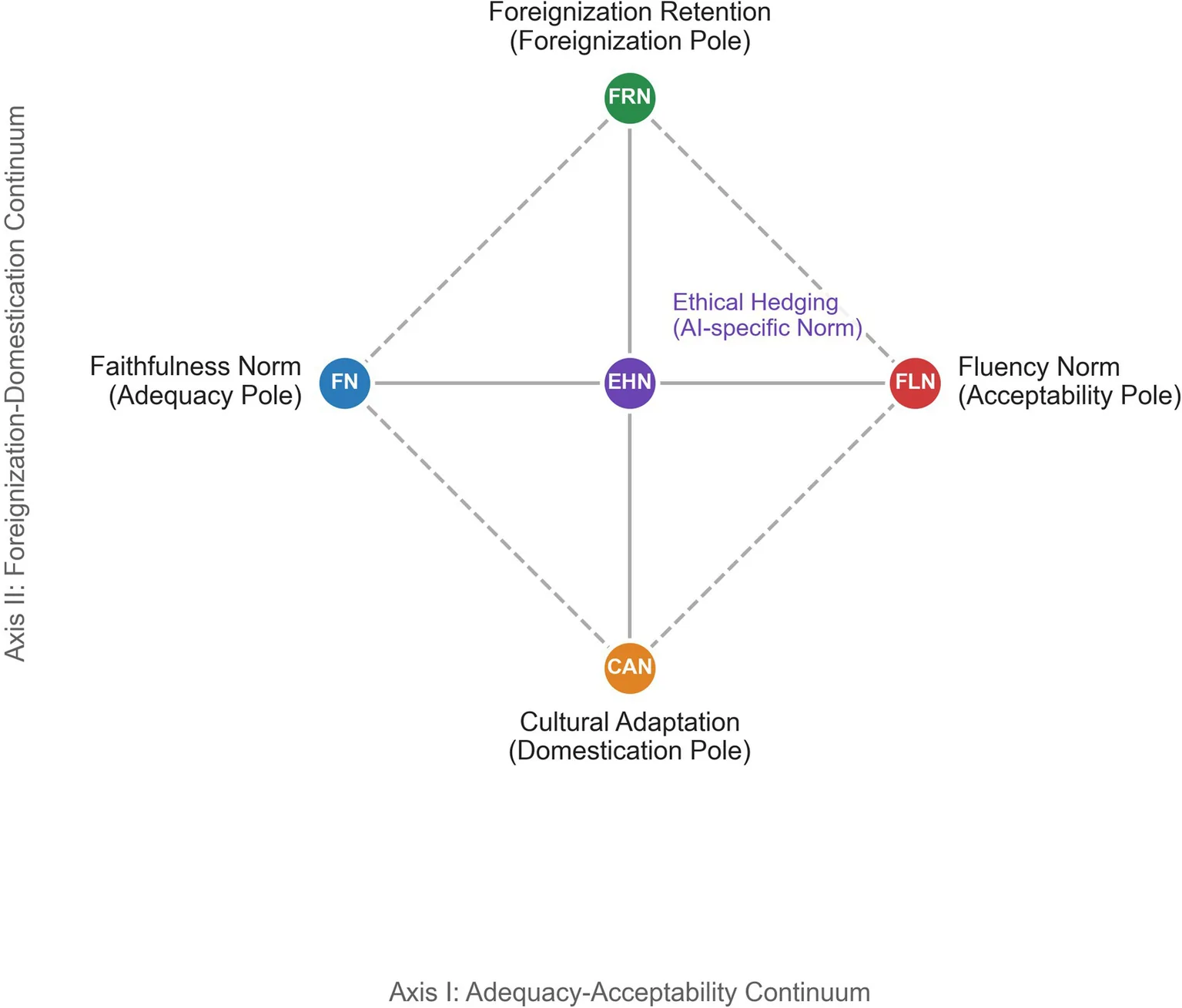
Tension structure of the Five-dimensional Translation Norm Orientation (TNO) Framework.

The eight candidate concepts from the systematic mapping were refined against three criteria—cross-theoretical traceability, behavioral observability, and activation under political discourse tension—and aggregated into the Translation Norm Orientations (TNO) five-dimensional framework (Figure 3).

#### 2.6.1 Faithfulness norm (FN)

Rooted in Toury’s (1995) adequacy prerequisite and Nida and Taber’s (1969) formal equivalence, FN demands high fidelity to source-language semantics, resisting meaning dilution through target-language reframing.

#### 2.6.2 Fluency norm (FLN)

Anchored in the acceptability pole (Toury, 1995) and domestication (Venuti, 2017), FLN captures the “fluency bias” internalized from dominant target-language training patterns (Jiang et al., 2023).

#### 2.6.3 Cultural adaptation norm (CAN)

Drawing on domestication (Venuti, 2017) and functional appropriateness (Nord, 2014), CAN substitutes culturally marked Chinese expressions with target-culture equivalents.

#### 2.6.4 Ethical hedging norm (EHN)

EHN captures an observable textual tendency toward semantic mitigation or dilution in translation options—a pattern that has been associated with RLHF-based safety alignment in prior work (Ouyang et al., 2022; Millière, 2025), though a mitigated rendering may equally reflect stylistic, register-based, or semantic-weakening choices unrelated to safety mechanisms. As operationalized in the FCTRT, EHN labels translation options that exhibit semantically diffuse, hedged renderings; it does not constitute a claim that such outputs are mechanistically produced by safety filters. Direct mechanistic attribution would require model-internal access beyond the scope of this behavioral study.

#### 2.6.5 Foreignization retention norm (FRN)

Aligned with Venuti’s (2017) foreignization strategy and Berman’s (1999) ethics of literal translation, FRN preserves source-language morphological distinctiveness, resisting assimilation by the dominant target culture and safeguarding the discursive autonomy of the source-cultural “other.”

## 3 Materials and methods

### 3.1 Overall research design

This study employs a controlled experimental design centered on the Forced-Choice Translation Ranking Task (FCTRT). By pre-specifying five candidate translation norms and requiring models to rank them within a unified semantic frame, the design isolates normative orientation from generative fluency—a confound that undermines *post-hoc* analyses of unconstrained outputs.

### 3.2 Source corpus construction

The stimulus corpus comprises 20 Chinese political and policy terms drawn from authentic official discourse contexts—including party and government documents, policy reports, diplomatic speeches, and official state media translations (see Supplementary material, Table 1)—each embedded in its authentic sentence frame rather than presented as a decontextualized lexical item. While the study focuses on term-level translation decisions, the full-sentence presentation preserves the pragmatic context in which each term functions. This domain was selected for three reasons. First, it concentrates normative tension at unusually high density; Valdeón and Li (2024) have called explicitly for quantitative designs in this area. Second, the structural asymmetries between Chinese and English political concepts routinely force consequential rewriting decisions (Munday, 2016), making the domain especially apt for surfacing systematic cross-model differences. Third, the substantial empirical literature on Chinese political discourse translation within Chinese academia ensures research feasibility (Wang, 2021). A further methodological rationale concerns construct validity: by drawing terms from authenticated official discourse contexts with traceable translation histories, the FCTRT options are grounded in documented professional practice rather than researcher intuition, reducing the risk that the instrument measures idiosyncratic lexical choices rather than genuine normative alternatives.

**Table 1:** FCTRT stimulus corpus: 20 test items.

Source term (Chinese)	English Gloss
	(FN rendering)
韬光养晦	Hiding one’s capabilities while biding one’s time
人类命运共同体	A community with a shared future for mankind
依法治国	Governing the country in accordance with law
一带一路	The Belt and Road
新质生产力	New quality productive forces
宣传	Propaganda
统一战线	The united front
维权	Rights protection
中国特色社会主义	Socialism with Chinese characteristics
生态文明	Ecological civilization
智能经济新形态	A new form of intelligent economy
具身智能	Embodied intelligence
算电协同	Computing-power and electricity coordination
国家低碳转型基金	National Low-Carbon Transition Fund
“内卷式”竞争	“Involution-type” competition
碳排放双控	Dual control of carbon emissions
智能原生	Intelligence-native
未来能源	Future energy
养老服务消费补贴	Elderly care service consumption subsidies
扩能提质	Expand capacity and improve quality

The corpus was assembled through a three-step double-blind procedure, conducted under the joint supervision of two doctoral supervisors who reviewed and confirmed each stage outcome. The procedure serves both as a corpus-assembly protocol and as the methodological guarantee of item-level validity:

Step 1 Candidate Generation. Two researchers independently screened items from three source domains: political governance discourse; 2026 government policy vocabulary, chosen to probe training-data cutoff effects (Zhang and Qin, 2025); and ethical discourse.

Step 2 Cross-Validation. Each candidate was required to satisfy three criteria: documented translation controversy; constructibility of all five TNO-aligned variants without semantic ambiguity; and inter-annotator *κ* ≥ 0.70 (Cohen, 1960). Items failing any criterion were excluded after independent verification by two doctoral-level translation scholars. To further strengthen external validity, one additional scholar holding a doctoral degree in Translation Studies and with no conflict of interest in this project conducted expert validation of the final 20 items: using a structured scoring rubric, this scholar independently confirmed that each option authentically operationalizes its assigned TNO dimension. The expert validation agreement rate with the dual-annotator coding is reported alongside the inter-annotator reliability statistics. The entire three-step procedure was conducted under the supervision of two doctoral supervisors who provided final confirmatory review, constituting an additional layer of procedural accountability.

Step 3 Final Determination. Twenty highly discriminative items were retained, evenly split between established political terms and recent policy vocabulary, minimizing researcher-induced sampling bias.

### 3.3 Stimulus material design

For each item, five translation options were constructed, one per TNO dimension, and embedded within a complete English sentence frame rather than presented in isolation. This full-sentential design overcomes context-free evaluation limitations (Huang et al., 2025) and leverages LLMs’ demonstrated sentence-level judgment capacity (Kocmi and Federmann, 2023).

Construct validity was verified via double-blind annotation: two independent Master’s-level annotators, blind to the hypotheses, assigned each option to a TNO dimension. Items with *κ* < 0.70 were revised and re-annotated; overall κ across all 100 options reached 0.79, exceeding the “substantial agreement” threshold (Landis and Koch, 1977). The annotation process was conducted under the oversight of two doctoral supervisors with expertise in translation studies, who reviewed the full annotation scheme and provided confirmatory adjudication for edge cases where the two annotators’ independent codes diverged after discussion.

Beyond dimensional assignment, the two annotators rated each option’s standalone translation acceptability on a 5-point scale (1 = implausible, 5 = fully acceptable). All 100 options scored ≥ 3.5 (*M* = 4.12, SD = 0.58), confirming comparable baseline plausibility across dimensions and mitigating the risk that rankings reflect stimulus quality rather than normative preference (see Table 2).

**Table 2:** Example: FCTRT stimulus for O-01 (韬光养晦).

Option	TNO Dim.	Translation of target term
A	FN	Hiding one’s capabilities while biding one’s time
B	FLN	Keeping a low profile
C	CAN	Strategic restraint
D	EHN	Maintaining a cautious and restrained international posture
E	FRN	tāoguāng yǎnghuì (keeping a low profile)

Three further safeguards address the concern that a unitary “translation quality” anchor may conflate quality with faithfulness. First, the prompt specifies “translation quality” without reference to accuracy, naturalness, or any normative criterion, maintaining non-directionality. Second, the discourse-type moderation effect reported in §5.1—where EHN rises for emerging terms and FLN rises for classic terms—constitutes internal evidence against tautology: if FN dominance were a pure anchor artifact, no systematic dimension-shifting across lexical categories would be expected. Third, the construct-validity data above confirm that all five options per item are independently plausible translations, ruling out the possibility that non-FN options were designed as implausibly poor distractors.

### 3.4 Model selection

Four industry-leading commercial LLMs were selected from each country, yielding eight models in total (Table 3). All model versions and API access timestamps were logged to ensure reproducibility (Laskar et al., 2024).

**Table 3:** List of representative large language models (LLMs).

Model name	Region	Developer/source
DeepSeek-V3.2	CN	DeepSeek
Kimi-2.5	CN	Moonshot AI
Qwen-3.5	CN	Alibaba
ERNIE 5.0	CN	Baidu
Claude Sonnet 4.6	US	Anthropic
GPT-5.3	US	OpenAI
Gemini-3.1-Pro	US	Google
Grok 4.2	US	xAI

### 3.5 Prompt design and data collection

Three prompt-design principles were enforced: (1) pure ranking without causal elicitation—explanation prompts were excluded to prevent rationale contamination; (2) neutrally framed instructions—"translation quality” served as the sole evaluation anchor; (3) bilingual parallel control—Chinese and English prompts with identical content neutralized language-of-prompting effects. The complete bilingual prompts for all 20 stimulus items, including the three permuted presentation orders, are provided in the Supplementary material.

Each item was presented across three rounds, with option order (A–E) permuted via a Latin square design (Gete and Etchegoyhen, 2024). All models were accessed via their official APIs between March and April 2026, using default parameter settings (temperature and sampling parameters as specified in each provider’s API defaults). Model versions are recorded in Table 3. Where a model returned an incomplete or malformed ranking (e.g., missing options, repeated ranks, or non-numeric responses), the response was flagged, the trial was re-administered once, and if still invalid, the item–model combination was excluded from that round’s analysis. No more than two such exclusions occurred across any single model. Kendall’s W (Kendall and Gibbons, 1962) and Friedman tests were computed for each item–model combination to assess ranking consistency; all item-level data were retained for primary analysis, with stability metrics reported as a separate diagnostic layer. The task enforces strict total ordering (ranks 1–5, no ties), maximizing inter-dimensional discrimination at the cost of artificial separation where models may regard options as equally acceptable.

### 3.6 Statistical analysis plan

For RQ1, mean rankings characterize overall normative preference patterns. For RQ2, Mann–Whitney U tests (with Welch’s t sensitivity check, BH-FDR correction, and Cliff’s *δ* effect size) probe between-group differences. For RQ3, unsupervised hierarchical clustering probes whether normative groupings track geopolitical or training-driven structures.

## 4 Results

### 4.1 Overall pattern of translation norm preferences

#### 4.1.1 Structural dominance of the faithfulness norm

**Figure 4:**
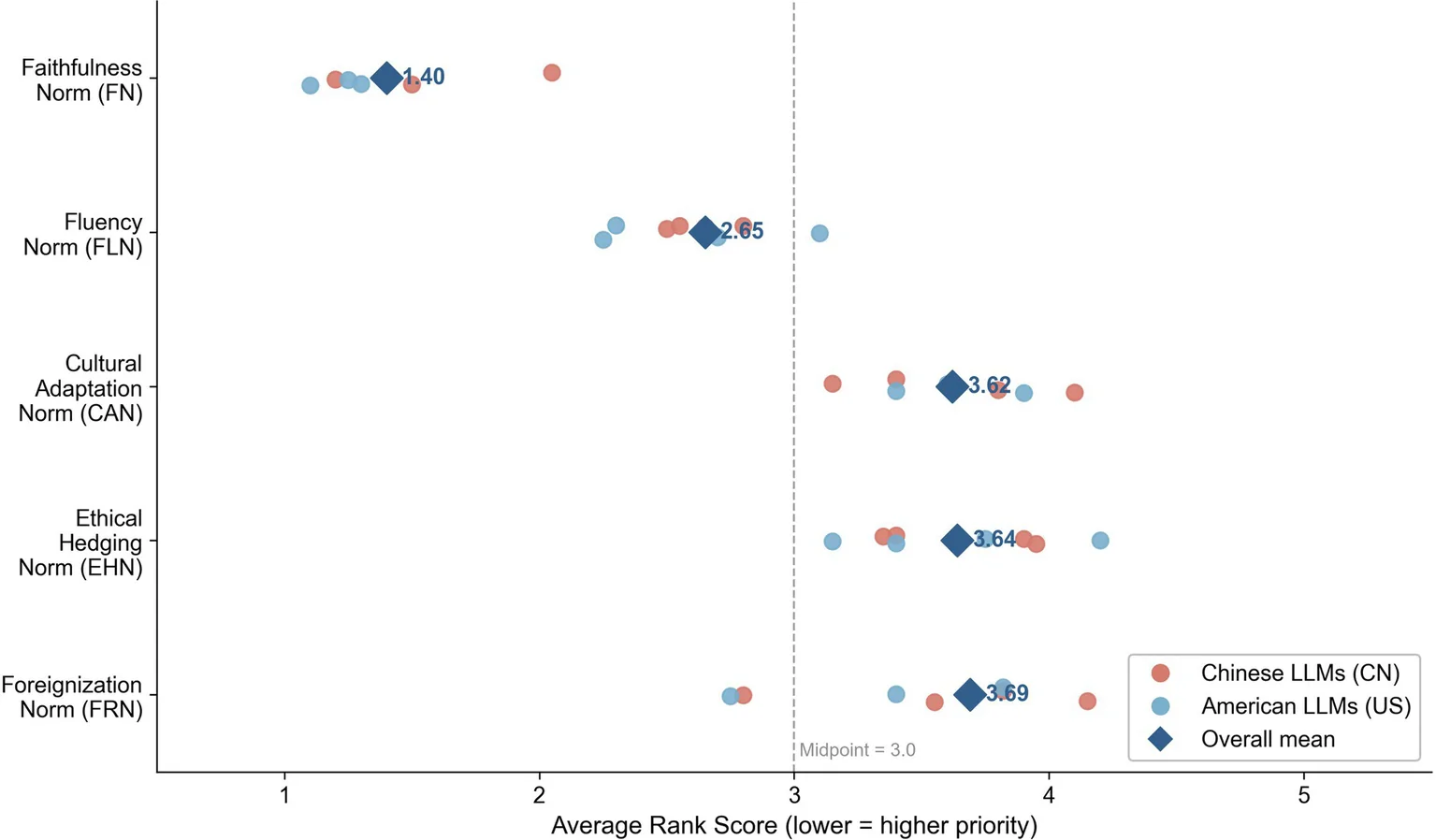
Overall priority distribution of translation norm orientations of eight LLMs.

Figure 4 presents the mean TNO rankings across the five dimensions for all eight LLMs over the 20 test items. The overall hierarchy is: FN (*M* = 1.40) > FLN (*M* = 2.65) > CAN (*M* = 3.62) ≈ EHN (*M* = 3.64) > FRN (*M* = 3.69), forming a clear gradient.

The most salient feature is FN’s zero-exception dominance: every model ranked it first. This consistency invites interpretation at two non-mutually-exclusive levels. At the level of disciplinary continuity, the formal-equivalence tradition (Toury, 1995; Nida and Taber, 1969) treats source-language semantic integrity as the primary constraint; pre-training corpora dominated by professional parallel texts already encode this faithfulness preference, so FN dominance partly reflects a statistical reproduction of embedded normative distributions (House, 2014). At the level of task design, the faithfulness option carries the lowest risk of semantic deviation and constitutes a “minimum-regret” choice whose priority may partly reflect risk aversion rather than deep normative internalization (Millière, 2025).

FLN occupies the second tier (*M* = 2.65), 1.25 points below FN. This runner-up position accords with the “fluency bias” documented by Jiang et al. (2023) and corroborates Baker’s normalization-as-universal prediction (Baker, 2018a; Halverson, 2003).

CAN, EHN, and FRN share a compressed low-priority zone (means 3.63–3.70). Although drawing from distinct theoretical traditions—domestication (Venuti, 2017), RLHF alignment (Ouyang et al., 2022), and foreignization ethics (Berman, 1999) respectively—all three are consistently deprioritized in the forced-ranking scenario of political discourse. Under conditions of heightened ideological sensitivity, culturally manipulative strategies of varying orientations appear to be systematically avoided as a class.

#### 4.1.2 Discourse-type moderation of normative priorities

**Figure 5:**
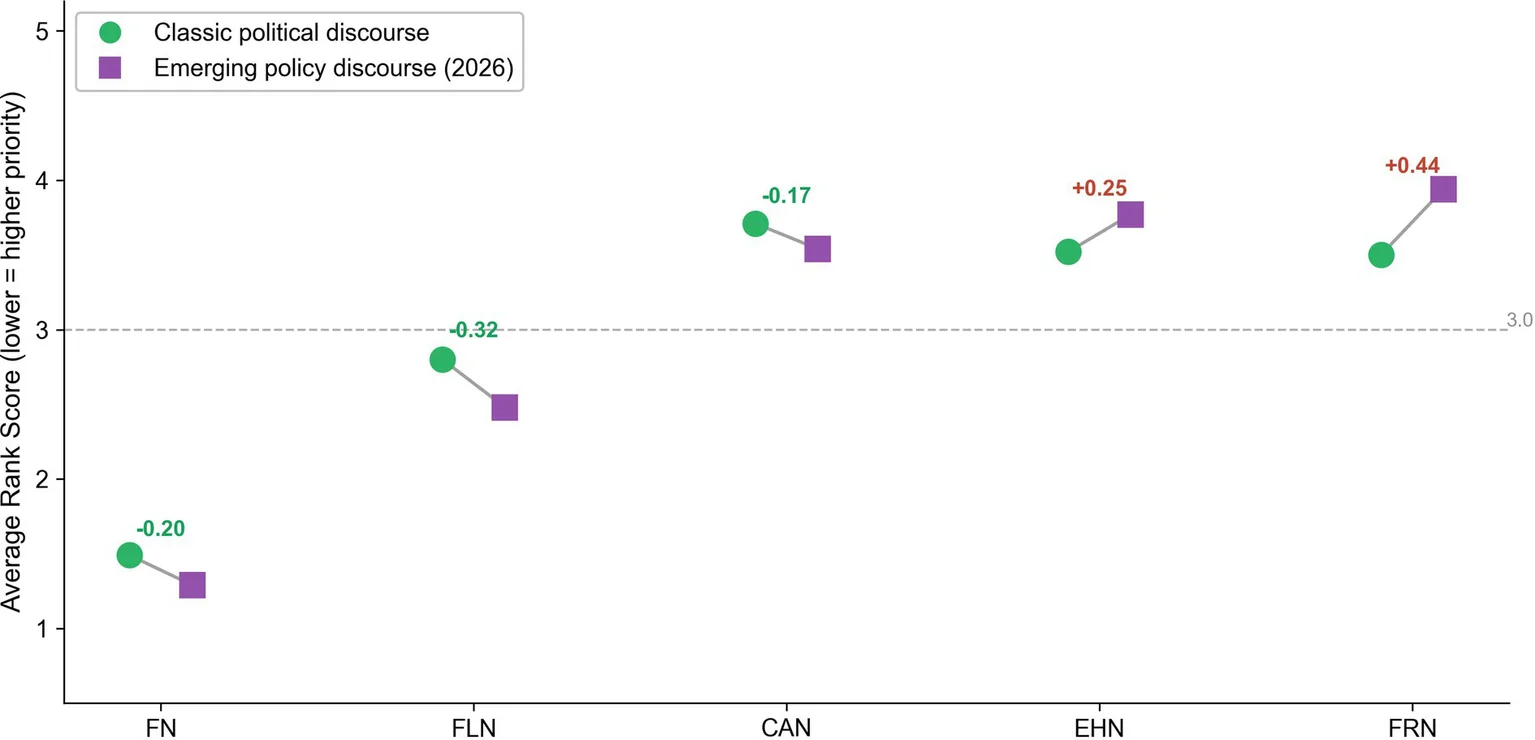
Moderating effect of discourse type on TNO priority.

Figure 5 disaggregates the overall pattern by lexical category, revealing a discourse-type moderation effect. Classic political terms (Category O) and emerging policy terms (Category N) diverge on two dimensions: EHN rises from *M* = 3.52 (O) to 3.77 (N), a + 0.25 shift toward hedging for emerging discourse; FLN moves oppositely, from *M* = 2.80 (O) to 2.48 (N), indicating stronger fluency preference for classic terms. FN remains dominant in both (*M* = 1.49 vs. 1.29).

This pattern admits a coherent explanation. Emerging policy vocabulary lacks established English equivalents; models can neither find mature domestication targets nor readily resort to transliteration, making semantically diffuse hedging options a comparatively accessible path and lifting EHN’s priority. Classic terms, by contrast, have accumulated stable equivalents through decades of bilingual practice, lowering the cost of fluency and elevating FLN—a discourse-type moderation consistent with Valdeón and Li’s (2024) observations.

#### 4.1.3 Ranking stability of translation norm preferences

**Figure 6:**
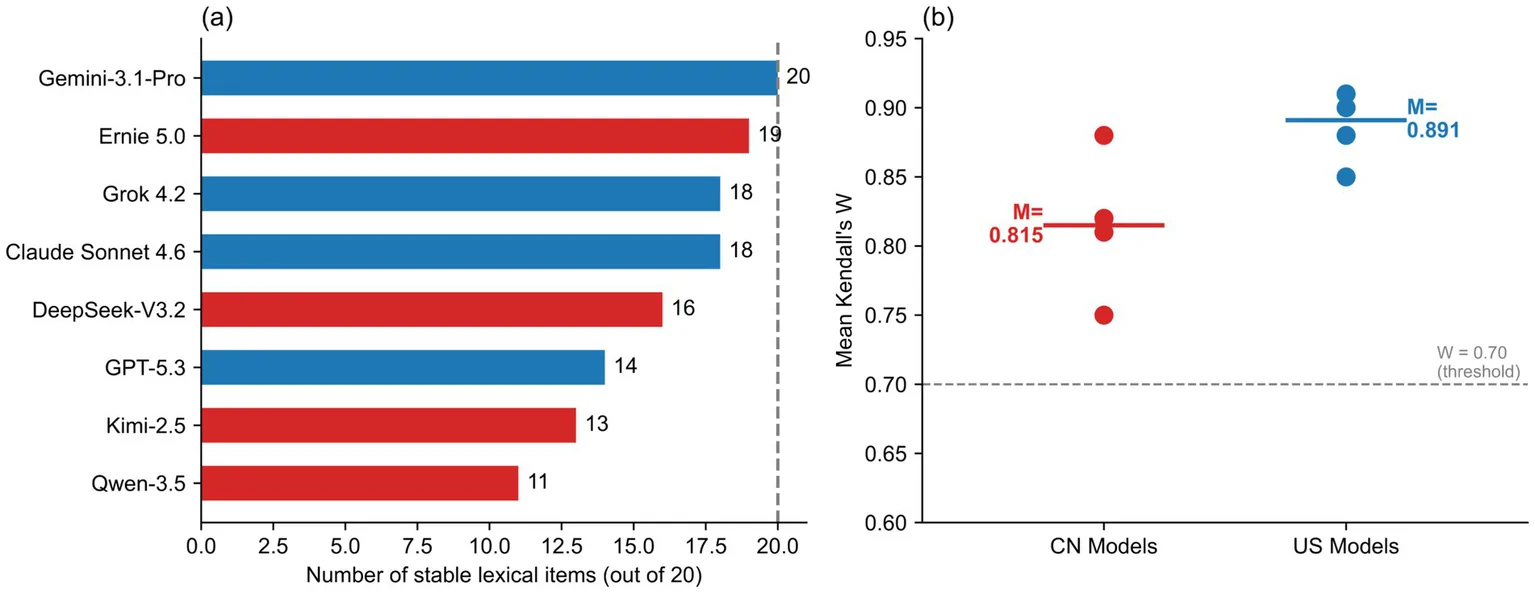
Overview of translation norm ranking stability across models. **(a)** Stable items per model. **(b)** Mean Kendall’s W by group.

Figure 6 presents item-level stability assessed via Kendall’s W and Friedman tests across three repeated trials. All eight models reached acceptable concordance levels (stable items per model: 11/20–20/20; mean W range: 0.750–0.913). The US group’s mean stability (M_W = 0.891) exceeded the CN group’s (M_W = 0.815) by a small margin, but the more informative contrast lies in dispersion: US models showed strong homogeneity, with every model yielding at least 14 stable items, whereas the CN group exhibited marked internal differentiation—a gap of eight items separates ERNIE 5.0 (19/20) from Qwen-3.5 (11/20). This asymmetry parallels the “internal heterogeneity” of Chinese models documented by Ma et al. (2026) in value-ranking stability.

Organizing unstable items (W < 0.70 or Friedman *p* ≥ 0.05) by lexical category reveals a discourse-dependent pattern. Several emerging policy terms (e.g., “involution-type competition,” “dual control of carbon emissions,” “elderly care service consumption subsidies”) recurrently showed inconsistent normative preferences across multiple models, whereas classic political terms (e.g., “tāoguāng yǎnghuì,” “the Belt and Road”) demonstrated higher overall stability. This pattern corroborates the discourse-type moderation reported above: emerging vocabulary, lacking established translation conventions, imposes greater uncertainty on normative judgments, manifesting as reduced cross-round consistency.

### 4.2 Between-group comparison

#### 4.2.1 Five-dimensional TNO comparison

**Figure 7:**
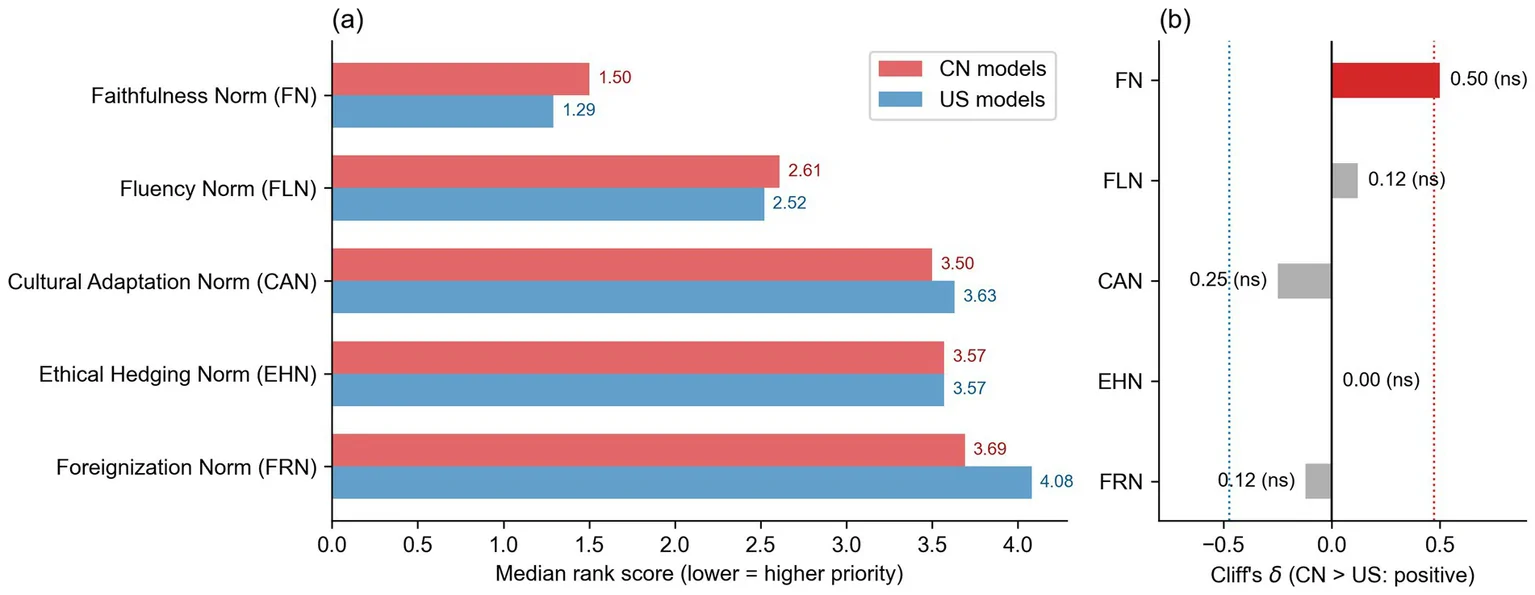
Inter-group comparison of TNO preferences between CN and US LLMs. **(a)** Median TNO scores: CN vs.US. **(b)** Effect size (Cliff’s δ).

With each model’s mean TNO scores across the 20 items as the unit of analysis, Mann–Whitney U tests (primary) and Welch’s t tests (sensitivity check) were conducted for each dimension between the CN group (*n* = 4) and US group (*n* = 4), with BH-FDR correction for multiple comparisons. None of the five dimensions reached statistical significance (BH-FDR-corrected *q* = 1.000 for all; Figure 7).

This convergence indicates that, in this exploratory sample, developer geography is not a decisive variable for translation norm preferences in political discourse. Technical homogenization offers a parsimonious explanation: mainstream LLMs undergo pre-training on English corpora of comparable scale and adopt closely aligned alignment frameworks, so that cultural origin is absorbed by shared technical infrastructure (Ma et al., 2026; Millière, 2025; Dahlgren Lindström et al., 2025).

#### 4.2.2 A directional signal in FN

Although non-significant, the FN dimension carries a medium-to-large effect size (Cliff’s *δ* = 0.50). The CN group’s median (1.498) is slightly higher than the US group’s (1.291), indicating a marginally stronger absolute FN preference among US models; crucially, the divergence concerns preference intensity rather than direction, as both groups rank FN first. *Post-hoc* sensitivity analysis indicates that with *n* = 4 per group, the Mann–Whitney U test reaches 80% power only for Cliff’s δ ≥ 0.90; the observed δ = 0.50 yields achieved power of approximately 0.18, so this signal is treated as exploratory. One plausible account is that Chinese models, exposed to richer Chinese political discourse during pre-training, lean marginally toward accessibility strategies, whereas US models default to faithful translation as the lower-risk path—consistent with prompt-language sensitivity (Zhong et al., 2024).

#### 4.2.3 Cross-group convergence on EHN

The inter-group difference on EHN was the smallest of any dimension: CN median = 3.566, US median = 3.571, Cliff’s δ = 0.000 (U = 8, *p* = 1.000). This near-identity initially appears to conflict with the expectation that divergent alignment regimes should produce divergent hedging tendencies. The resolution lies in distinguishing EHN as an active normative strategy from safety filtering as a boundary constraint. The FCTRT measures the former; when all options fall within the compliant range, safety filters are not triggered and preferences revert to translation-quality judgments. The low EHN priority likely reflects that actively choosing a diluted rendering conflicts with the default criterion of faithfulness. Whether this pattern is linked to safety-alignment mechanisms or simply to stylistic preferences cannot be determined from ranking data alone.

#### 4.2.4 Clustering structure and descriptive model profiles

**Figure 8:**
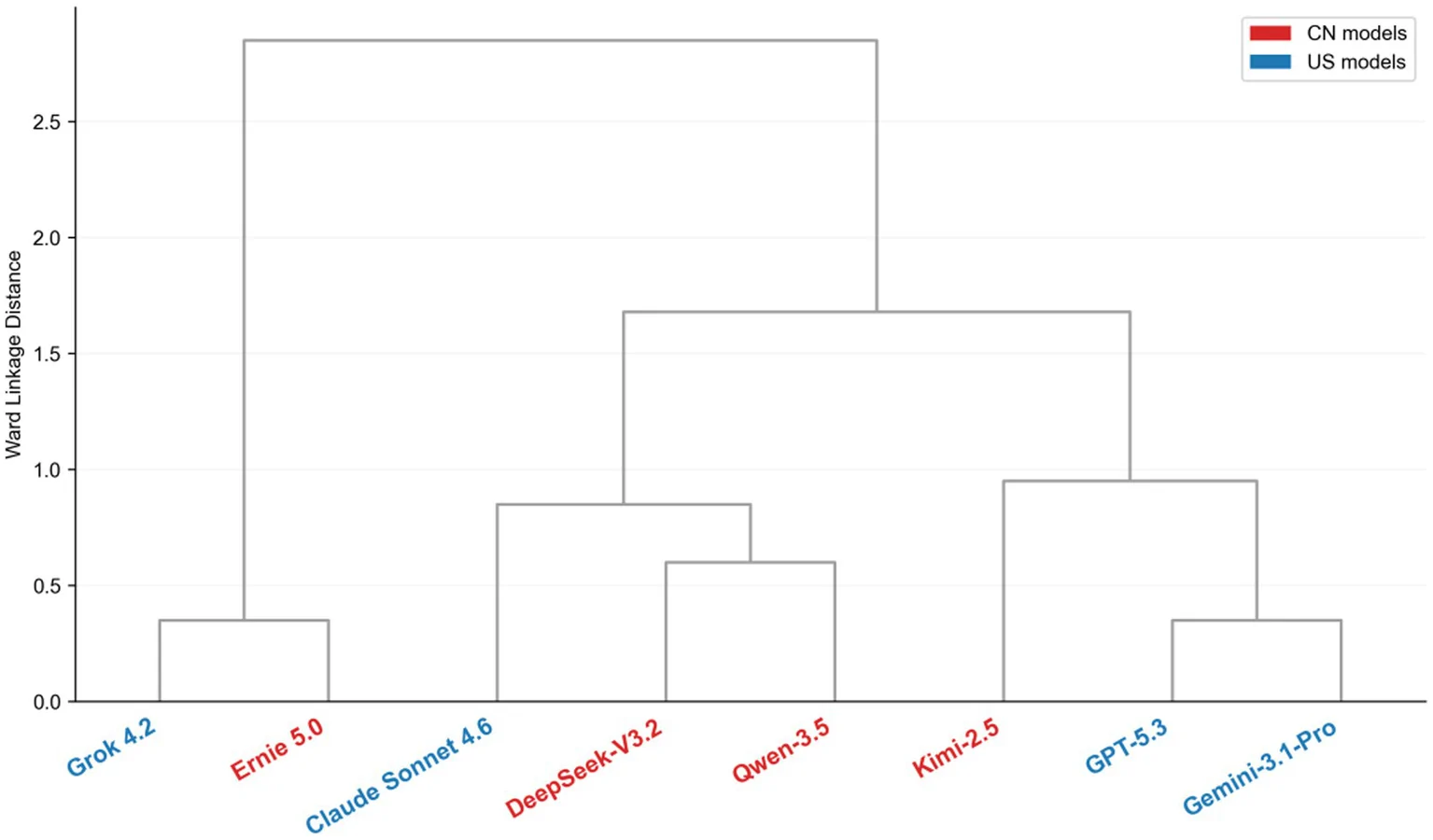
Hierarchical clustering dendrogram of LLMs based on TNO preference vectors.

Using each model’s mean TNO scores across the 20 items as feature vectors, unsupervised hierarchical clustering was conducted with Ward’s linkage and Euclidean distance. The resulting dendrogram (Figure 8) reveals that clusters do not align neatly along CN–US geopolitical boundaries; instead, prominent cross-national mixed clusters emerge. Three cross-national mixed clusters emerge (see Figure 8 for full membership), with the tightest pair—Grok 4.2 (US) and ERNIE 5.0 (CN)—merging at the lowest distance level.

Inter-model FRN variance far exceeds that on the other four dimensions, making foreignization tolerance the primary differentiation axis. Grok 4.2 and ERNIE 5.0 share low FRN means (2.72, 2.79), exhibiting openness to source-culture markers across national lines; GPT-5.3 and Gemini-3.1-Pro display the highest FRN means (4.62, 4.65), reflecting strong foreignization avoidance—yet Grok 4.2, also a US model, sits far from them, confirming that differentiation tracks foreignization tolerance rather than geography (Naous and Xu, 2025).

#### 4.2.5 Four descriptive model profiles

**Figure 9:**
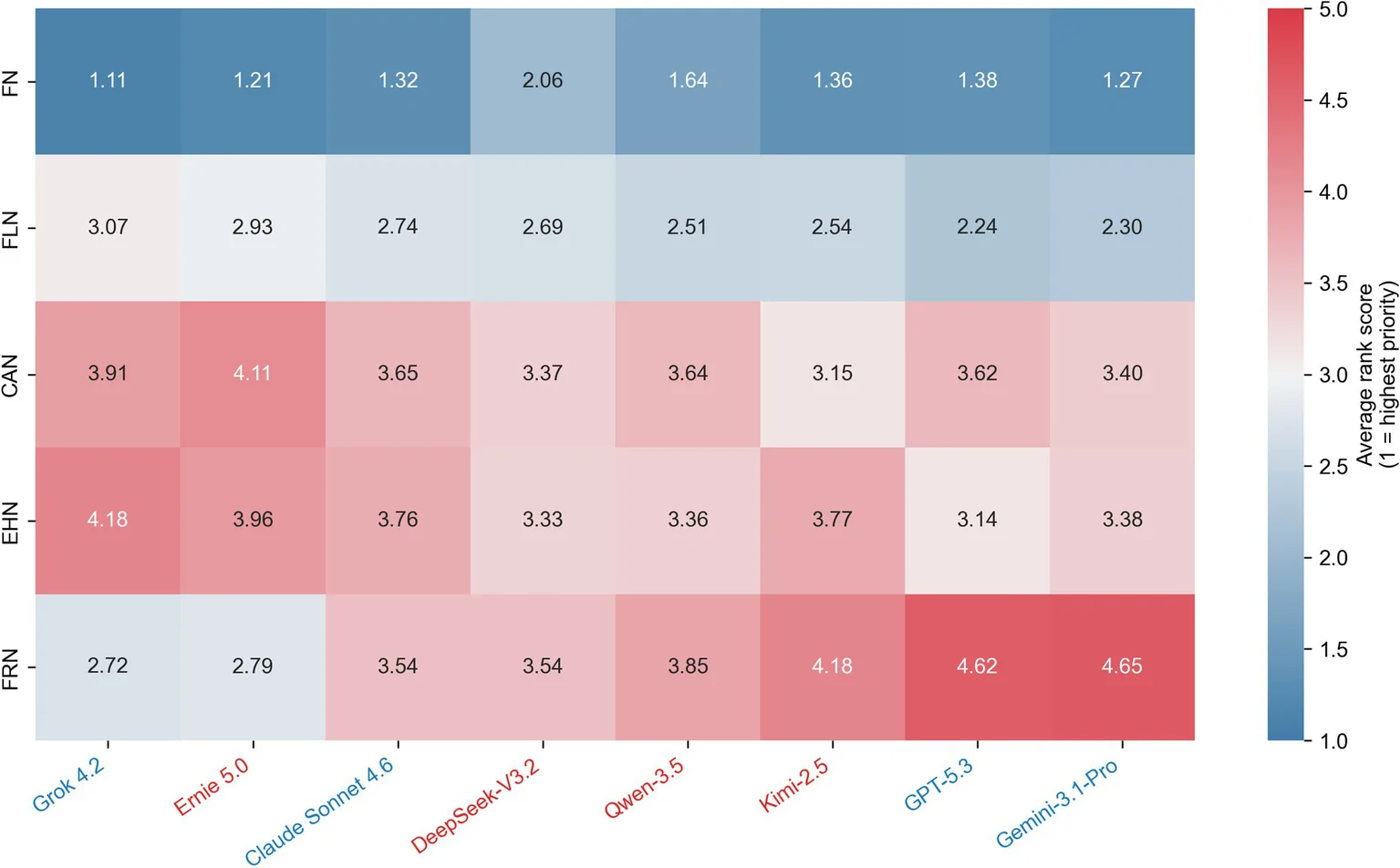
Clustered heatmap of TNO rankings across all models.

Given the small sample of eight models, the clusters below should be interpreted as preliminary descriptive profiles rather than stable taxonomic archetypes; bootstrap resampling in future larger-sample replications would be needed to assess cluster stability. The clustered heatmap (Figure 9) delineates four provisional archetypes:

1. Form-Preserving. Representatives: Grok 4.2 (US), ERNIE 5.0 (CN). Absolute FN priority (1.11, 1.21) with the lowest FRN (2.72, 2.79), tending toward close semantic translation that incorporates source-culture markers rather than replacing them.
2. Faithful-Evasive. Representatives: GPT-5.3 (US), Gemini-3.1-Pro (US), Kimi-2.5 (CN). FN first (1.27–1.38) with strongest FRN avoidance (4.18–4.65), favoring culturally legible renderings that actively assimilate source-culture markers.
3. Faithful-Fluent Balanced. Representatives: Claude Sonnet 4.6 (US), Qwen-3.5 (CN). FN priority (1.32, 1.64) with FLN close behind (2.74, 2.51); the smallest overall variance yields the most predictable profile.
4. Faithful-Adaptive. Representative: DeepSeek-V3.2 (CN). FN mean (2.06), the weakest in the sample; the most balanced FN–FLN distribution, actively seeking target-language adaptability within a faithfulness frame.

#### 4.2.6 Intra-group homogeneity

**Figure 10:**
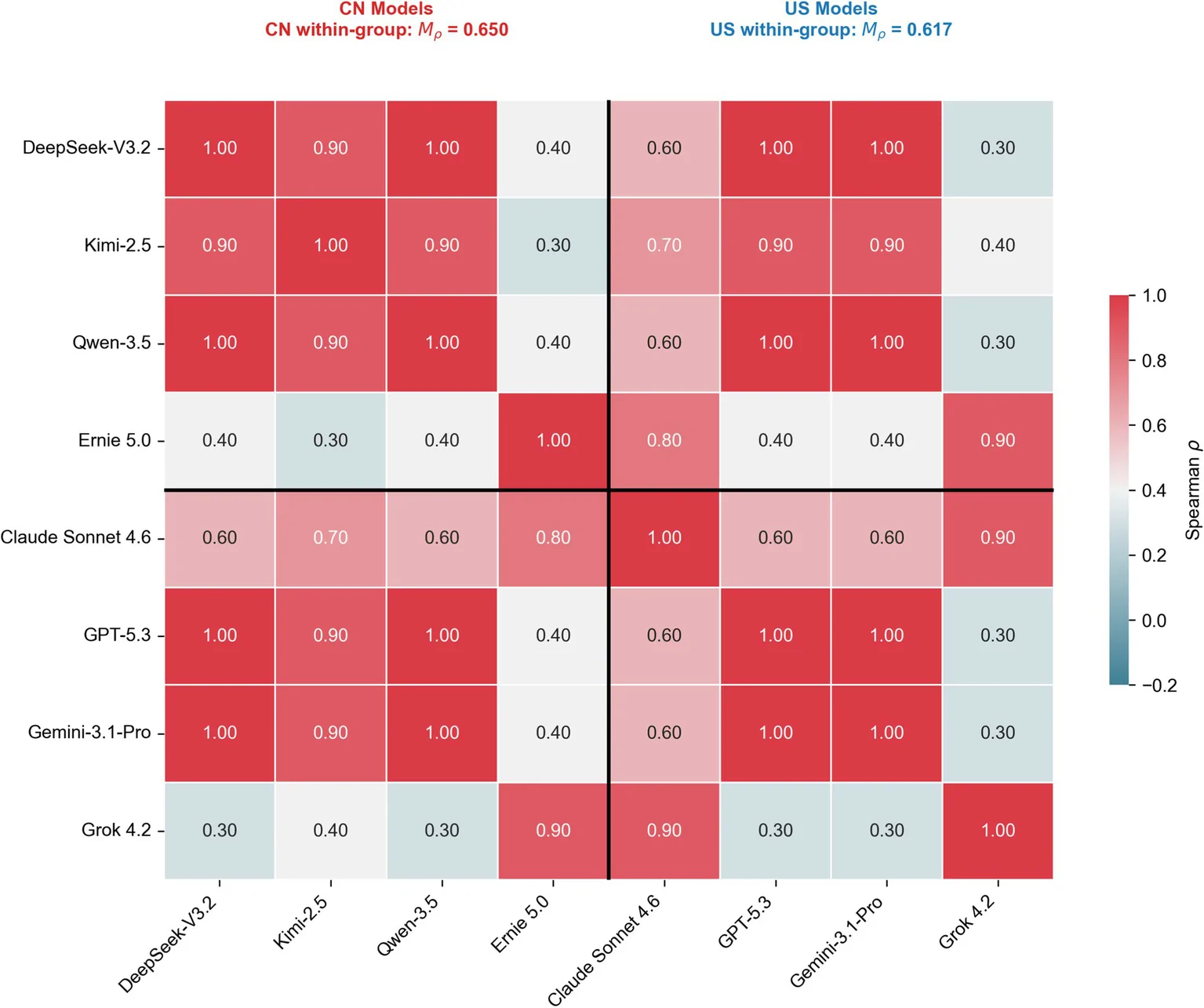
Pairwise Spearman correlation heatmap of TNO preference vectors across all models.

The Spearman correlation heatmap (Figure 10) confirms a shared normative structure (all pairwise *ρ* > 0.5; CN M_ρ = 0.650, US M_ρ = 0.617), indicating negligible national-origin influence on intra-group cohesion.

## 5 Discussion

### 5.1 Methodological scope and caveats

The present study is best understood as an exploratory investigation of observable ranking tendencies under controlled FCTRT conditions. The FCTRT measures ranking preferences under controlled conditions but does not capture tendencies that surface in free generation—where Wen and Tian (2024) observed implicit EHN activation structurally suppressed by the ranking paradigm. FCTRT-type instruments and free-generation analyses should thus be treated as complementary. The present sample (*n* = 4 per group) constrains statistical power, and treating model-level means as independent observations does not account for item-level nesting; a mixed-effects framework treating items as random effects nested within models would yield more precise estimates. The medium FN effect size (Cliff’s *δ* = 0.50) warrants replication with larger model sets and item-level modeling. Moreover, recent work on rating indeterminacy demonstrates that forced-choice protocols can misrepresent preferences when the option space is under-specified (Guerdan et al., 2026); a multi-label paradigm permitting tied ranks or “equally acceptable” judgments would complement the FCTRT’s ordinal strictness. We acknowledge that forced-ranking with a unitary “quality” anchor cannot fully rule out the possibility that models implicitly equate quality with semantic fidelity; dimension-specific anchors (e.g., “cultural appropriateness”) would serve as a valuable robustness check in future designs. Relatedly, the unitary “translation quality” anchor does not specify target register; testing whether register-specific instructions (e.g., journalistic vs. academic) shift dimension rankings would further strengthen discriminant validity.

Given the sample of eight models, all findings should be read as descriptive patterns for this specific model set rather than generalizable conclusions about Chinese or American LLMs as classes. Differences or similarities observed here may not replicate with a different or expanded model sample.

### 5.2 Implications for translation tool selection

Three practical implications follow. First, although FN dominance indicates that LLMs broadly preserve source-language semantic structure, Faithful-Evasive models (e.g., GPT-5.3, Gemini-3.1-Pro) achieve fidelity while rejecting source-culture markers—producing implicit cultural smoothing—and elevated EHN activation for emerging policy vocabulary calls for supplementary human review of policy neologisms. Second, normative archetype predicts translational behavior far more reliably than country of origin, suggesting archetype-based criteria for cross-border tool selection.

### 5.3 Limitations and future work

The present study has six limitations that bound the interpretation of findings. First, the model sample (*n* = 8, *n* = 4 per national group) is too small for inferential statistical power; the reported effect sizes and clustering patterns should be treated as descriptive rather than confirmatory. Second, the stimulus corpus is confined to 20 terms from Chinese official political and policy discourse; generalizability to other domains (legal, medical, literary) or other language pairs cannot be assumed without replication. Third, although the FCTRT construction involved double-blind annotation, doctoral-level expert validation, and supervisory confirmation, rankings were not cross-validated against human professional translator rankings or automated quality metrics; establishing convergent validity through such data triangulation remains a priority for future research. Fourth, the FCTRT employs a unitary ‘translation quality’ anchor; future designs with dimension-specific anchors (e.g., ‘cultural appropriateness’, ‘target-audience intelligibility’) would test whether normative priorities are criterion-sensitive. Fifth, the static snapshot design cannot capture normative drift; model versions updated after the April 2026 access window may yield different preference profiles. Sixth, because FCTRT is a forced-choice paradigm, normative tendencies that emerge only in free generation—where competing options do not constrain output—remain undetected by the present design.

#### 5.3.1 Six research directions follow from these limitations

(1) Scale replication: extending the sample to 20–30 models per national group, including open-source and instruction-tuned variants, would provide the statistical power needed to test between-group differences inferentially and assess cluster stability through bootstrap resampling. (2) Cross-domain extension: applying the FCTRT to legal, medical, and literary corpora would test whether FN dominance is domain-general or domain-sensitive, and whether political discourse represents an unusually high normative-tension context. (3) Human-LLM triangulation: administering the same FCTRT items to experienced professional translators would establish human baseline rankings and enable direct convergent validity assessment—the data triangulation identified as a current limitation. (4) Longitudinal monitoring: re-administering the FCTRT to the same models at six-month intervals would track normative drift across model version updates, safety-alignment changes, and new training corpora. (5) Multi-criterion designs: replacing the unitary quality anchor with explicit sub-criteria anchors in parallel FCTRT administrations would reveal whether normative priorities are anchor-sensitive, strengthening discriminant validity. (6) Free-generation pairing: combining FCTRT ranking tasks with open-ended translation of the same items would test whether forced-choice preferences predict generative behavior, a key assumption of the instrument’s ecological validity that current data cannot address.

## 6 Conclusion

The three research questions posed at the outset can now be addressed directly. RQ1—whether LLMs exhibit stable, observable ranking tendencies in translating Chinese political and policy terms—is answered affirmatively: all eight models consistently assigned Faithfulness Norm (FN) the highest priority across all 20 items and all three permuted rounds, confirming robust and cross-nationally shared ranking behavior under controlled FCTRT conditions. RQ2—whether Chinese and American LLMs differ in normative ranking preferences—is answered largely negatively: Mann–Whitney U rank-sum tests returned non-significant between-group differences on all five norm dimensions (all *p* > 0.05), with near-zero effect sizes on EHN (Cliff’s *δ* = 0.000) and a medium effect on FN (Cliff’s δ = 0.50) that is directionally consistent but not inferentially reliable at the current sample size, indicating that developer geography is not a systematic discriminator in this exploratory sample. RQ3—whether normative profiles align with geopolitical boundaries—is answered negatively: hierarchical clustering yielded four transnational descriptive model profiles organized primarily by foreignization tolerance (FRN as the primary differentiation axis), with cross-national model pairings appearing in each cluster.

Using the TNO framework and FCTRT, this exploratory study assessed observable ranking tendencies in eight Chinese and American LLMs across 20 ideologically sensitive Chinese political and policy terms drawn from authentic official discourse contexts. The Faithfulness Norm occupies a structurally dominant position; Cultural Adaptation, Ethical Hedging, and Foreignization Retention cluster in a shared low-priority zone. No statistically significant cross-national differences emerged; clustering yielded four transnational preliminary descriptive profiles differentiated by foreignization tolerance rather than geography. Current LLMs converge as faithful translation instruments across national origins, while preserving discourse-sensitive variation that merits continued scrutiny.

## Data Availability

The datasets presented in this study can be found in online repositories. The names of the repository/repositories and accession number(s) can be found in the article/Supplementary material.
