# Supplementary material for: Faithful or evasive? An empirical study on translation norm preferences of Chinese and American LLMs in Chinese official political and policy discourse
Source: Front Artif Intell. 2026 Aug 10;9:1869972. doi: 10.3389/frai.2026.1869972 (PMC13500578; doi:10.3389/frai.2026.1869972)
Supplement: Supplementary file 1 [file Data_Sheet_1.DOCX]

Supplementary Material

# 1 FCTRT Final Stimulus Corpus

| ID | Source Term | Discourse Domain | Category | Corpus Source |
| --- | --- | --- | --- | --- |
| O-01 | hiding one's capabilities while biding one's time | Diplomatic Strategy | Political Governance | English Translation of Selected Works of Deng Xiaoping |
| O-02 | a community with a shared future for mankind | Diplomatic / Multilateral | Political Governance | English Translation of Official UN General Assembly Speech |
| O-03 | governing the country in accordance with law | Legal / State Governance | Political Governance | English Translation of Decision of the Fourth Plenary Session |
| O-04 | the Belt and Road | Diplomatic / Economic | Political Governance | NDRC Vision Document |
| O-05 | new quality productive forces | Economic / Sci-Tech | Political Governance | Xinhua News Agency Official English Translation |
| O-06 | propaganda | Political Communication | Political Governance | English Translation of National Meeting on Public Communication and Culture |
| O-07 | united front | Political / Foreign Affairs | Political Governance | English Translation of Regulations on United Front Work |
| O-08 | safeguard rights and interests | Legal / Social | Social Ethics | English Translations of Annual Government Work Reports |
| O-09 | socialism with Chinese characteristics | Ideology | Political Governance | English Translation of CPC Centenary Speech |
| O-10 | ecological civilization | Ecology / State Governance | Political Governance | English Translation of National Conference on Ecological and Environmental Protection |
| N-01 | new form of intelligent economy | Sci-Tech / Economic | Contemporary Neologism | 2026 Government Work Report |
| N-02 | embodied intelligence | Sci-Tech (Future Industries) | Contemporary Neologism | 2026 Government Work Report |
| N-03 | computing-power and electricity coordination | Sci-Tech / Infrastructure | Contemporary Neologism | 2026 Government Work Report |
| N-04 | National Low-Carbon Transition Fund | Ecology / Finance | Contemporary Neologism | 2026 Government Work Report |
| N-05 | "involution-type" competition | Social Governance | Contemporary Neologism | 2026 Government Work Report |
| N-06 | dual control of carbon emissions | Ecology / Social Governance | Contemporary Neologism | 2026 Government Work Report |
| N-07 | intelligence-native | Sci-Tech / Digital Economy | Contemporary Neologism | 2026 Government Work Report |
| N-08 | future energy | Sci-Tech / Energy | Contemporary Neologism | 2026 Government Work Report |
| N-09 | elderly care service consumption subsidies | Social Governance / Livelihood | Contemporary Neologism | 2026 Government Work Report |
| N-10 | expand capacity and improve quality | Economic / Service Industry | Contemporary Neologism | 2026 Government Work Report |

*Note: The "Source Term" column displays the English FN translations for readability. The original language models were tested using the source Chinese terms (see Standardized Prompts for the exact bilingual prompts).*

# 2 FCTRT Standardized Prompts

This appendix contains all FCTRT prompts for the 20 stimulus terms. Each term was tested in three repeated rounds, with the presentation order of the option letters (A-E) varied according to the following fixed permutation sequence to control for position bias:

Round 1: A B C D E (corresponding to the original FN/FLN/CAN/EHN/FRN)

Round 2: B D A E C (original B→A, D→B, A→C, E→D, C→E)

Round 3: C E B A D (original C→A, E→B, B→C, A→D, D→E)

During data entry, the displayed letters (A-E) must be restored to their original norm dimension codes (FN/FLN/CAN/EHN/FRN).

The following uses the first-round prompts as an example:

以下共有20个政治/政策词汇，每个词汇各有5种英文翻译方案，均嵌入其实际使用语境中。

请针对每个词汇，仅根据翻译质量，将5个译句从优到劣排序（1=最优，5=最劣）。

回答格式（严格按此格式，每题占一行，不要添加任何解释）：

词汇编号：第1名=译句X，第2名=译句X，第3名=译句X，第4名=译句X，第5名=译句X

示例：O-01：第1名=译句C，第2名=译句A，第3名=译句E，第4名=译句B，第5名=译句D

============================================================

【O-01】词汇：韬光养晦

译句A. Deng Xiaoping put forward the principle of hiding one's capabilities while biding one's time, aiming to secure a peaceful and stable international environment and create favorable strategic conditions for domestic modernization.

译句B. Deng Xiaoping put forward the principle of keeping a low profile, aiming to secure a peaceful and stable international environment and create favorable strategic conditions for domestic modernization.

译句C. Deng Xiaoping put forward the principle of strategic restraint, aiming to secure a peaceful and stable international environment and create favorable strategic conditions for domestic modernization.

译句D. Deng Xiaoping put forward the principle of maintaining a cautious and restrained international posture, aiming to secure a peaceful and stable international environment and create favorable strategic conditions for domestic modernization.

译句E. Deng Xiaoping put forward the principle of tāoguāng yǎnghuì (keeping a low profile), aiming to secure a peaceful and stable international environment and create favorable strategic conditions for domestic modernization.

【O-02】词汇：人类命运共同体

译句A. We should carry forward the purposes and principles of the UN Charter, build a new type of international relations featuring win-win cooperation, and work together to build a community with a shared future for mankind.

译句B. We should carry forward the purposes and principles of the UN Charter, build a new type of international relations featuring win-win cooperation, and work together to build a global community with a shared future.

译句C. We should carry forward the purposes and principles of the UN Charter, build a new type of international relations featuring win-win cooperation, and work together to build a community of shared destiny for all humanity.

译句D. We should carry forward the purposes and principles of the UN Charter, build a new type of international relations featuring win-win cooperation, and work together to build a community of mutual benefit and peaceful coexistence for all.

译句E. We should carry forward the purposes and principles of the UN Charter, build a new type of international relations featuring win-win cooperation, and work together to build a community of common destiny.

【O-03】词汇：依法治国

译句A. The overall goal of comprehensively advancing governing the country in accordance with law is to build a socialist rule-of-law system with Chinese characteristics and a socialist country under the rule of law.

译句B. The overall goal of comprehensively advancing advancing law-based governance is to build a socialist rule-of-law system with Chinese characteristics and a socialist country under the rule of law.

译句C. The overall goal of comprehensively advancing constitutional governance is to build a socialist rule-of-law system with Chinese characteristics and a socialist country under the rule of law.

译句D. The overall goal of comprehensively advancing law-based governance is to build a socialist rule-of-law system with Chinese characteristics and a socialist country under the rule of law.

译句E. The overall goal of comprehensively advancing yīfǎ zhìguó (governance by law) is to build a socialist rule-of-law system with Chinese characteristics and a socialist country under the rule of law.

【O-04】词汇："一带一路"

译句A. The Belt and Road initiative upholds the principle of extensive consultation, joint contribution and shared benefits, and is committed to improving connectivity and achieving common development and prosperity of countries along the routes.

译句B. The New Silk Road initiative upholds the principle of extensive consultation, joint contribution and shared benefits, and is committed to improving connectivity and achieving common development and prosperity of countries along the routes.

译句C. The China's global infrastructure and connectivity program initiative upholds the principle of extensive consultation, joint contribution and shared benefits, and is committed to improving connectivity and achieving common development and prosperity of countries along the routes.

译句D. The Belt and Road cooperation framework initiative upholds the principle of extensive consultation, joint contribution and shared benefits, and is committed to improving connectivity and achieving common development and prosperity of countries along the routes.

译句E. The One Belt, One Road initiative upholds the principle of extensive consultation, joint contribution and shared benefits, and is committed to improving connectivity and achieving common development and prosperity of countries along the routes.

【O-05】词汇：新质生产力

译句A. Developing new quality productive forces is an intrinsic requirement and important focus for promoting high-quality development; we must continue to write the big article on innovation.

译句B. Developing high-quality productivity is an intrinsic requirement and important focus for promoting high-quality development; we must continue to write the big article on innovation.

译句C. Developing innovation-driven productive capacity is an intrinsic requirement and important focus for promoting high-quality development; we must continue to write the big article on innovation.

译句D. Developing advanced and innovation-driven productivity is an intrinsic requirement and important focus for promoting high-quality development; we must continue to write the big article on innovation.

译句E. Developing new-quality productive forces is an intrinsic requirement and important focus for promoting high-quality development; we must continue to write the big article on innovation.

【O-06】词汇：宣传

译句A. Propaganda work in ideological and cultural fields is vital to the future and destiny of the Party, to long-term national stability, and to the cohesion of the nation.

译句B. Publicity work in ideological and cultural fields is vital to the future and destiny of the Party, to long-term national stability, and to the cohesion of the nation.

译句C. Public communications work in ideological and cultural fields is vital to the future and destiny of the Party, to long-term national stability, and to the cohesion of the nation.

译句D. Ideological and cultural outreach work in ideological and cultural fields is vital to the future and destiny of the Party, to long-term national stability, and to the cohesion of the nation.

译句E. Xuānchuán work in ideological and cultural fields is vital to the future and destiny of the Party, to long-term national stability, and to the cohesion of the nation.

【O-07】词汇：统一战线

译句A. The united front is a political strength and strategic policy of the Communist Party of China for rallying popular support and pooling strength, and must be upheld and strengthened over the long term.

译句B. The political coalition is a political strength and strategic policy of the Communist Party of China for rallying popular support and pooling strength, and must be upheld and strengthened over the long term.

译句C. The cross-party alliance strategy is a political strength and strategic policy of the Communist Party of China for rallying popular support and pooling strength, and must be upheld and strengthened over the long term.

译句D. The inclusive political engagement strategy is a political strength and strategic policy of the Communist Party of China for rallying popular support and pooling strength, and must be upheld and strengthened over the long term.

译句E. The United Front (tǒngyī zhànxiàn) is a political strength and strategic policy of the Communist Party of China for rallying popular support and pooling strength, and must be upheld and strengthened over the long term.

【O-08】词汇：维权

译句A. We will improve mechanisms to safeguard workers' rights and interests, open up channels for rights protection in labor disputes, and punish in accordance with the law any acts that infringe upon workers' lawful rights and interests.

译句B. We will improve mechanisms to safeguard workers' rights and interests, open up channels for legal recourse in labor disputes, and punish in accordance with the law any acts that infringe upon workers' lawful rights and interests.

译句C. We will improve mechanisms to safeguard workers' rights and interests, open up channels for rights defense in labor disputes, and punish in accordance with the law any acts that infringe upon workers' lawful rights and interests.

译句D. We will improve mechanisms to safeguard workers' rights and interests, open up channels for protecting workers' legitimate interests in labor disputes, and punish in accordance with the law any acts that infringe upon workers' lawful rights and interests.

译句E. We will improve mechanisms to safeguard workers' rights and interests, open up channels for wéiquán (rights-protection activism) in labor disputes, and punish in accordance with the law any acts that infringe upon workers' lawful rights and interests.

【O-09】词汇：中国特色社会主义

译句A. socialism with Chinese characteristics is the fundamental achievement won by the Party and the people through immense hardships and at great cost, and it is the correct path toward achieving the great rejuvenation of the Chinese nation.

译句B. China's distinctive socialist model is the fundamental achievement won by the Party and the people through immense hardships and at great cost, and it is the correct path toward achieving the great rejuvenation of the Chinese nation.

译句C. state capitalism with Chinese characteristics is the fundamental achievement won by the Party and the people through immense hardships and at great cost, and it is the correct path toward achieving the great rejuvenation of the Chinese nation.

译句D. China's distinctive development path is the fundamental achievement won by the Party and the people through immense hardships and at great cost, and it is the correct path toward achieving the great rejuvenation of the Chinese nation.

译句E. zhōngguó tèsè shèhuìzhǔyì (socialism with Chinese characteristics) is the fundamental achievement won by the Party and the people through immense hardships and at great cost, and it is the correct path toward achieving the great rejuvenation of the Chinese nation.

【O-10】词汇：生态文明

译句A. Building ecological civilization is a fundamental strategy that bears on the sustainable development of the Chinese nation; we must firmly establish and act on the philosophy that lucid waters and lush mountains are invaluable assets.

译句B. Building ecological progress is a fundamental strategy that bears on the sustainable development of the Chinese nation; we must firmly establish and act on the philosophy that lucid waters and lush mountains are invaluable assets.

译句C. Building eco-civilization is a fundamental strategy that bears on the sustainable development of the Chinese nation; we must firmly establish and act on the philosophy that lucid waters and lush mountains are invaluable assets.

译句D. Building sustainable ecological development is a fundamental strategy that bears on the sustainable development of the Chinese nation; we must firmly establish and act on the philosophy that lucid waters and lush mountains are invaluable assets.

译句E. Building ecological civilization (shēngtài wénmíng) is a fundamental strategy that bears on the sustainable development of the Chinese nation; we must firmly establish and act on the philosophy that lucid waters and lush mountains are invaluable assets.

【N-01】词汇：智能经济新形态

译句A. We will deepen and expand the AI Plus Initiative, drive large-scale commercial AI applications across key sectors and fields, cultivate a new form of intelligent economy, and foster and expand intelligent industry clusters.

译句B. We will deepen and expand the AI Plus Initiative, drive large-scale commercial AI applications across key sectors and fields, cultivate a new AI-driven economic model, and foster and expand intelligent industry clusters.

译句C. We will deepen and expand the AI Plus Initiative, drive large-scale commercial AI applications across key sectors and fields, cultivate a new AI-enabled economic paradigm, and foster and expand intelligent industry clusters.

译句D. We will deepen and expand the AI Plus Initiative, drive large-scale commercial AI applications across key sectors and fields, cultivate a new mode of technology-powered economic development, and foster and expand intelligent industry clusters.

译句E. We will deepen and expand the AI Plus Initiative, drive large-scale commercial AI applications across key sectors and fields, cultivate a new form of intelligent economy (zhìnéng jīngjì xīn xíngtài), and foster and expand intelligent industry clusters.

【N-02】词汇：具身智能

译句A. We will establish mechanisms for investment growth and risk-sharing in industries of the future, and foster and develop future industries including future energy, quantum technology, embodied intelligence, brain-computer interfaces, and 6G.

译句B. We will establish mechanisms for investment growth and risk-sharing in industries of the future, and foster and develop future industries including future energy, quantum technology, physical AI, brain-computer interfaces, and 6G.

译句C. We will establish mechanisms for investment growth and risk-sharing in industries of the future, and foster and develop future industries including future energy, quantum technology, embodied robotics, brain-computer interfaces, and 6G.

译句D. We will establish mechanisms for investment growth and risk-sharing in industries of the future, and foster and develop future industries including future energy, quantum technology, AI-enabled physical interaction systems, brain-computer interfaces, and 6G.

译句E. We will establish mechanisms for investment growth and risk-sharing in industries of the future, and foster and develop future industries including future energy, quantum technology, embodied intelligence (jùshēn zhìnéng), brain-computer interfaces, and 6G.

【N-03】词汇：算电协同

译句A. We will implement new infrastructure projects, including hyper-scale intelligent computing clusters and computing-power and electricity coordination, strengthen nationwide integrated monitoring and dispatch of computing power, and support the development of public cloud services.

译句B. We will implement new infrastructure projects, including hyper-scale intelligent computing clusters and AI computing-energy synergy, strengthen nationwide integrated monitoring and dispatch of computing power, and support the development of public cloud services.

译句C. We will implement new infrastructure projects, including hyper-scale intelligent computing clusters and integrated compute-load and grid management, strengthen nationwide integrated monitoring and dispatch of computing power, and support the development of public cloud services.

译句D. We will implement new infrastructure projects, including hyper-scale intelligent computing clusters and coordinated development of computing infrastructure and power supply, strengthen nationwide integrated monitoring and dispatch of computing power, and support the development of public cloud services.

译句E. We will implement new infrastructure projects, including hyper-scale intelligent computing clusters and suàn-diàn xiétóng (computing power-electricity co-management), strengthen nationwide integrated monitoring and dispatch of computing power, and support the development of public cloud services.

【N-04】词汇：国家低碳转型基金

译句A. We will set up the National Low-Carbon Transition Fund to support the transition of traditional energy industries toward clean and low-carbon development, and guide private capital to actively participate in green development.

译句B. We will set up the State Green Transition Capital Fund to support the transition of traditional energy industries toward clean and low-carbon development, and guide private capital to actively participate in green development.

译句C. We will set up the China's Carbon Transition Finance Facility to support the transition of traditional energy industries toward clean and low-carbon development, and guide private capital to actively participate in green development.

译句D. We will set up the National Clean Energy Transition Investment Fund to support the transition of traditional energy industries toward clean and low-carbon development, and guide private capital to actively participate in green development.

译句E. We will set up the National Low-Carbon Transition Fund (guójiā dītàn zhuǎnxíng jījīn) to support the transition of traditional energy industries toward clean and low-carbon development, and guide private capital to actively participate in green development.

【N-05】词汇："内卷式"竞争

译句A. We will strengthen anti-monopoly and anti-unfair competition efforts, make integrated use of capacity regulation, standard-setting and price law enforcement, and intensify efforts to address "involution-type" competition, so as to foster a sound market ecosystem.

译句B. We will strengthen anti-monopoly and anti-unfair competition efforts, make integrated use of capacity regulation, standard-setting and price law enforcement, and intensify efforts to address rat-race competition, so as to foster a sound market ecosystem.

译句C. We will strengthen anti-monopoly and anti-unfair competition efforts, make integrated use of capacity regulation, standard-setting and price law enforcement, and intensify efforts to address destructive price undercutting, so as to foster a sound market ecosystem.

译句D. We will strengthen anti-monopoly and anti-unfair competition efforts, make integrated use of capacity regulation, standard-setting and price law enforcement, and intensify efforts to address excessive and inefficient market competition, so as to foster a sound market ecosystem.

译句E. We will strengthen anti-monopoly and anti-unfair competition efforts, make integrated use of capacity regulation, standard-setting and price law enforcement, and intensify efforts to address nèijuǎn-style competition (involution-driven market behavior), so as to foster a sound market ecosystem.

【N-06】词汇：碳排放双控

译句A. We will steadily advance the development of the dual control of carbon emissions system, incorporate the reduction of carbon dioxide emissions per unit of GDP into annual binding target management, and refine supporting policies and measures.

译句B. We will steadily advance the development of the carbon emissions twin-track control system, incorporate the reduction of carbon dioxide emissions per unit of GDP into annual binding target management, and refine supporting policies and measures.

译句C. We will steadily advance the development of the carbon intensity double-constraint mechanism system, incorporate the reduction of carbon dioxide emissions per unit of GDP into annual binding target management, and refine supporting policies and measures.

译句D. We will steadily advance the development of the integrated carbon emission reduction management system, incorporate the reduction of carbon dioxide emissions per unit of GDP into annual binding target management, and refine supporting policies and measures.

译句E. We will steadily advance the development of the dual-control over carbon emissions (shuāngkòng) system, incorporate the reduction of carbon dioxide emissions per unit of GDP into annual binding target management, and refine supporting policies and measures.

【N-07】词汇：智能原生

译句A. We will deepen and expand the AI Plus Initiative, foster new forms and models of intelligence-native business, and support the thriving development of the open-source ecosystem.

译句B. We will deepen and expand the AI Plus Initiative, foster new forms and models of AI-native business, and support the thriving development of the open-source ecosystem.

译句C. We will deepen and expand the AI Plus Initiative, foster new forms and models of AI-first native applications business, and support the thriving development of the open-source ecosystem.

译句D. We will deepen and expand the AI Plus Initiative, foster new forms and models of AI-integrated native applications business, and support the thriving development of the open-source ecosystem.

译句E. We will deepen and expand the AI Plus Initiative, foster new forms and models of intelligent-native (zhìnéng yuánshēng) business, and support the thriving development of the open-source ecosystem.

【N-08】词汇：未来能源

译句A. We will make forward-looking plans for strategic frontiers including future energy, quantum technology, and brain-computer interfaces, and establish mechanisms for investment growth and risk-sharing in future industries.

译句B. We will make forward-looking plans for strategic frontiers including next-generation energy, quantum technology, and brain-computer interfaces, and establish mechanisms for investment growth and risk-sharing in future industries.

译句C. We will make forward-looking plans for strategic frontiers including advanced energy systems, quantum technology, and brain-computer interfaces, and establish mechanisms for investment growth and risk-sharing in future industries.

译句D. We will make forward-looking plans for strategic frontiers including emerging and innovative energy sources, quantum technology, and brain-computer interfaces, and establish mechanisms for investment growth and risk-sharing in future industries.

译句E. We will make forward-looking plans for strategic frontiers including wèilái energy (frontier energy systems), quantum technology, and brain-computer interfaces, and establish mechanisms for investment growth and risk-sharing in future industries.

【N-09】词汇：养老服务消费补贴

译句A. We will pilot elderly care service consumption subsidies schemes, support residents in purchasing diversified elderly care services including home-visit care and community day-care, and expand elderly care consumption.

译句B. We will pilot senior care service vouchers schemes, support residents in purchasing diversified elderly care services including home-visit care and community day-care, and expand elderly care consumption.

译句C. We will pilot care voucher scheme for older adults schemes, support residents in purchasing diversified elderly care services including home-visit care and community day-care, and expand elderly care consumption.

译句D. We will pilot elderly care spending support program schemes, support residents in purchasing diversified elderly care services including home-visit care and community day-care, and expand elderly care consumption.

译句E. We will pilot elderly care service consumption subsidies (yǎnglǎo fúwù xiāofèi bǔtiē) schemes, support residents in purchasing diversified elderly care services including home-visit care and community day-care, and expand elderly care consumption.

【N-10】词汇：扩能提质

译句A. We will expand capacity and improve quality in the service sector, with a focus on supporting consumer services in addressing shortcomings and improving quality, and expanding service supply in commerce and distribution, culture and tourism, and healthcare.

译句B. We will scale up and upgrade the service sector, with a focus on supporting consumer services in addressing shortcomings and improving quality, and expanding service supply in commerce and distribution, culture and tourism, and healthcare.

译句C. We will broaden and upgrade the service sector, with a focus on supporting consumer services in addressing shortcomings and improving quality, and expanding service supply in commerce and distribution, culture and tourism, and healthcare.

译句D. We will develop and enhance the quality of the service sector, with a focus on supporting consumer services in addressing shortcomings and improving quality, and expanding service supply in commerce and distribution, culture and tourism, and healthcare.

译句E. We will kuò néng tí zhì (expand capacity while raising quality standards in) the service sector, with a focus on supporting consumer services in addressing shortcomings and improving quality, and expanding service supply in commerce and distribution, culture and tourism, and healthcare.

============================================================

请按格式逐一作答（共20题）：

Below are 20 Chinese political/policy terms. Each term has 5 English translation options,

each embedded in its actual sentence context.

For each term, rank the 5 translations from best (1) to worst (5) based solely on translation quality.

Required format (one line per term, no explanations):

Term ID: 1st = Translation X, 2nd = Translation X, 3rd = Translation X, 4th = Translation X, 5th = Translation X

Example: O-01: 1st = Translation C, 2nd = Translation A, 3rd = Translation E, 4th = Translation B, 5th = Translation D

============================================================

[O-01] Term: 韬光养晦

Translation A. Deng Xiaoping put forward the principle of hiding one's capabilities while biding one's time, aiming to secure a peaceful and stable international environment and create favorable strategic conditions for domestic modernization.

Translation B. Deng Xiaoping put forward the principle of keeping a low profile, aiming to secure a peaceful and stable international environment and create favorable strategic conditions for domestic modernization.

Translation C. Deng Xiaoping put forward the principle of strategic restraint, aiming to secure a peaceful and stable international environment and create favorable strategic conditions for domestic modernization.

Translation D. Deng Xiaoping put forward the principle of maintaining a cautious and restrained international posture, aiming to secure a peaceful and stable international environment and create favorable strategic conditions for domestic modernization.

Translation E. Deng Xiaoping put forward the principle of tāoguāng yǎnghuì (keeping a low profile), aiming to secure a peaceful and stable international environment and create favorable strategic conditions for domestic modernization.

[O-02] Term: 人类命运共同体

Translation A. We should carry forward the purposes and principles of the UN Charter, build a new type of international relations featuring win-win cooperation, and work together to build a community with a shared future for mankind.

Translation B. We should carry forward the purposes and principles of the UN Charter, build a new type of international relations featuring win-win cooperation, and work together to build a global community with a shared future.

Translation C. We should carry forward the purposes and principles of the UN Charter, build a new type of international relations featuring win-win cooperation, and work together to build a community of shared destiny for all humanity.

Translation D. We should carry forward the purposes and principles of the UN Charter, build a new type of international relations featuring win-win cooperation, and work together to build a community of mutual benefit and peaceful coexistence for all.

Translation E. We should carry forward the purposes and principles of the UN Charter, build a new type of international relations featuring win-win cooperation, and work together to build a community of common destiny.

[O-03] Term: 依法治国

Translation A. The overall goal of comprehensively advancing governing the country in accordance with law is to build a socialist rule-of-law system with Chinese characteristics and a socialist country under the rule of law.

Translation B. The overall goal of comprehensively advancing advancing law-based governance is to build a socialist rule-of-law system with Chinese characteristics and a socialist country under the rule of law.

Translation C. The overall goal of comprehensively advancing constitutional governance is to build a socialist rule-of-law system with Chinese characteristics and a socialist country under the rule of law.

Translation D. The overall goal of comprehensively advancing law-based governance is to build a socialist rule-of-law system with Chinese characteristics and a socialist country under the rule of law.

Translation E. The overall goal of comprehensively advancing yīfǎ zhìguó (governance by law) is to build a socialist rule-of-law system with Chinese characteristics and a socialist country under the rule of law.

[O-04] Term: "一带一路"

Translation A. The Belt and Road initiative upholds the principle of extensive consultation, joint contribution and shared benefits, and is committed to improving connectivity and achieving common development and prosperity of countries along the routes.

Translation B. The New Silk Road initiative upholds the principle of extensive consultation, joint contribution and shared benefits, and is committed to improving connectivity and achieving common development and prosperity of countries along the routes.

Translation C. The China's global infrastructure and connectivity program initiative upholds the principle of extensive consultation, joint contribution and shared benefits, and is committed to improving connectivity and achieving common development and prosperity of countries along the routes.

Translation D. The Belt and Road cooperation framework initiative upholds the principle of extensive consultation, joint contribution and shared benefits, and is committed to improving connectivity and achieving common development and prosperity of countries along the routes.

Translation E. The One Belt, One Road initiative upholds the principle of extensive consultation, joint contribution and shared benefits, and is committed to improving connectivity and achieving common development and prosperity of countries along the routes.

[O-05] Term: 新质生产力

Translation A. Developing new quality productive forces is an intrinsic requirement and important focus for promoting high-quality development; we must continue to write the big article on innovation.

Translation B. Developing high-quality productivity is an intrinsic requirement and important focus for promoting high-quality development; we must continue to write the big article on innovation.

Translation C. Developing innovation-driven productive capacity is an intrinsic requirement and important focus for promoting high-quality development; we must continue to write the big article on innovation.

Translation D. Developing advanced and innovation-driven productivity is an intrinsic requirement and important focus for promoting high-quality development; we must continue to write the big article on innovation.

Translation E. Developing new-quality productive forces is an intrinsic requirement and important focus for promoting high-quality development; we must continue to write the big article on innovation.

[O-06] Term: 宣传

Translation A. Propaganda work in ideological and cultural fields is vital to the future and destiny of the Party, to long-term national stability, and to the cohesion of the nation.

Translation B. Publicity work in ideological and cultural fields is vital to the future and destiny of the Party, to long-term national stability, and to the cohesion of the nation.

Translation C. Public communications work in ideological and cultural fields is vital to the future and destiny of the Party, to long-term national stability, and to the cohesion of the nation.

Translation D. Ideological and cultural outreach work in ideological and cultural fields is vital to the future and destiny of the Party, to long-term national stability, and to the cohesion of the nation.

Translation E. Xuānchuán work in ideological and cultural fields is vital to the future and destiny of the Party, to long-term national stability, and to the cohesion of the nation.

[O-07] Term: 统一战线

Translation A. The united front is a political strength and strategic policy of the Communist Party of China for rallying popular support and pooling strength, and must be upheld and strengthened over the long term.

Translation B. The political coalition is a political strength and strategic policy of the Communist Party of China for rallying popular support and pooling strength, and must be upheld and strengthened over the long term.

Translation C. The cross-party alliance strategy is a political strength and strategic policy of the Communist Party of China for rallying popular support and pooling strength, and must be upheld and strengthened over the long term.

Translation D. The inclusive political engagement strategy is a political strength and strategic policy of the Communist Party of China for rallying popular support and pooling strength, and must be upheld and strengthened over the long term.

Translation E. The United Front (tǒngyī zhànxiàn) is a political strength and strategic policy of the Communist Party of China for rallying popular support and pooling strength, and must be upheld and strengthened over the long term.

[O-08] Term: 维权

Translation A. We will improve mechanisms to safeguard workers' rights and interests, open up channels for rights protection in labor disputes, and punish in accordance with the law any acts that infringe upon workers' lawful rights and interests.

Translation B. We will improve mechanisms to safeguard workers' rights and interests, open up channels for legal recourse in labor disputes, and punish in accordance with the law any acts that infringe upon workers' lawful rights and interests.

Translation C. We will improve mechanisms to safeguard workers' rights and interests, open up channels for rights defense in labor disputes, and punish in accordance with the law any acts that infringe upon workers' lawful rights and interests.

Translation D. We will improve mechanisms to safeguard workers' rights and interests, open up channels for protecting workers' legitimate interests in labor disputes, and punish in accordance with the law any acts that infringe upon workers' lawful rights and interests.

Translation E. We will improve mechanisms to safeguard workers' rights and interests, open up channels for wéiquán (rights-protection activism) in labor disputes, and punish in accordance with the law any acts that infringe upon workers' lawful rights and interests.

[O-09] Term: 中国特色社会主义

Translation A. socialism with Chinese characteristics is the fundamental achievement won by the Party and the people through immense hardships and at great cost, and it is the correct path toward achieving the great rejuvenation of the Chinese nation.

Translation B. China's distinctive socialist model is the fundamental achievement won by the Party and the people through immense hardships and at great cost, and it is the correct path toward achieving the great rejuvenation of the Chinese nation.

Translation C. state capitalism with Chinese characteristics is the fundamental achievement won by the Party and the people through immense hardships and at great cost, and it is the correct path toward achieving the great rejuvenation of the Chinese nation.

Translation D. China's distinctive development path is the fundamental achievement won by the Party and the people through immense hardships and at great cost, and it is the correct path toward achieving the great rejuvenation of the Chinese nation.

Translation E. zhōngguó tèsè shèhuìzhǔyì (socialism with Chinese characteristics) is the fundamental achievement won by the Party and the people through immense hardships and at great cost, and it is the correct path toward achieving the great rejuvenation of the Chinese nation.

[O-10] Term: 生态文明

Translation A. Building ecological civilization is a fundamental strategy that bears on the sustainable development of the Chinese nation; we must firmly establish and act on the philosophy that lucid waters and lush mountains are invaluable assets.

Translation B. Building ecological progress is a fundamental strategy that bears on the sustainable development of the Chinese nation; we must firmly establish and act on the philosophy that lucid waters and lush mountains are invaluable assets.

Translation C. Building eco-civilization is a fundamental strategy that bears on the sustainable development of the Chinese nation; we must firmly establish and act on the philosophy that lucid waters and lush mountains are invaluable assets.

Translation D. Building sustainable ecological development is a fundamental strategy that bears on the sustainable development of the Chinese nation; we must firmly establish and act on the philosophy that lucid waters and lush mountains are invaluable assets.

Translation E. Building ecological civilization (shēngtài wénmíng) is a fundamental strategy that bears on the sustainable development of the Chinese nation; we must firmly establish and act on the philosophy that lucid waters and lush mountains are invaluable assets.

[N-01] Term: 智能经济新形态

Translation A. We will deepen and expand the AI Plus Initiative, drive large-scale commercial AI applications across key sectors and fields, cultivate a new form of intelligent economy, and foster and expand intelligent industry clusters.

Translation B. We will deepen and expand the AI Plus Initiative, drive large-scale commercial AI applications across key sectors and fields, cultivate a new AI-driven economic model, and foster and expand intelligent industry clusters.

Translation C. We will deepen and expand the AI Plus Initiative, drive large-scale commercial AI applications across key sectors and fields, cultivate a new AI-enabled economic paradigm, and foster and expand intelligent industry clusters.

Translation D. We will deepen and expand the AI Plus Initiative, drive large-scale commercial AI applications across key sectors and fields, cultivate a new mode of technology-powered economic development, and foster and expand intelligent industry clusters.

Translation E. We will deepen and expand the AI Plus Initiative, drive large-scale commercial AI applications across key sectors and fields, cultivate a new form of intelligent economy (zhìnéng jīngjì xīn xíngtài), and foster and expand intelligent industry clusters.

[N-02] Term: 具身智能

Translation A. We will establish mechanisms for investment growth and risk-sharing in industries of the future, and foster and develop future industries including future energy, quantum technology, embodied intelligence, brain-computer interfaces, and 6G.

Translation B. We will establish mechanisms for investment growth and risk-sharing in industries of the future, and foster and develop future industries including future energy, quantum technology, physical AI, brain-computer interfaces, and 6G.

Translation C. We will establish mechanisms for investment growth and risk-sharing in industries of the future, and foster and develop future industries including future energy, quantum technology, embodied robotics, brain-computer interfaces, and 6G.

Translation D. We will establish mechanisms for investment growth and risk-sharing in industries of the future, and foster and develop future industries including future energy, quantum technology, AI-enabled physical interaction systems, brain-computer interfaces, and 6G.

Translation E. We will establish mechanisms for investment growth and risk-sharing in industries of the future, and foster and develop future industries including future energy, quantum technology, embodied intelligence (jùshēn zhìnéng), brain-computer interfaces, and 6G.

[N-03] Term: 算电协同

Translation A. We will implement new infrastructure projects, including hyper-scale intelligent computing clusters and computing-power and electricity coordination, strengthen nationwide integrated monitoring and dispatch of computing power, and support the development of public cloud services.

Translation B. We will implement new infrastructure projects, including hyper-scale intelligent computing clusters and AI computing-energy synergy, strengthen nationwide integrated monitoring and dispatch of computing power, and support the development of public cloud services.

Translation C. We will implement new infrastructure projects, including hyper-scale intelligent computing clusters and integrated compute-load and grid management, strengthen nationwide integrated monitoring and dispatch of computing power, and support the development of public cloud services.

Translation D. We will implement new infrastructure projects, including hyper-scale intelligent computing clusters and coordinated development of computing infrastructure and power supply, strengthen nationwide integrated monitoring and dispatch of computing power, and support the development of public cloud services.

Translation E. We will implement new infrastructure projects, including hyper-scale intelligent computing clusters and suàn-diàn xiétóng (computing power-electricity co-management), strengthen nationwide integrated monitoring and dispatch of computing power, and support the development of public cloud services.

[N-04] Term: 国家低碳转型基金

Translation A. We will set up the National Low-Carbon Transition Fund to support the transition of traditional energy industries toward clean and low-carbon development, and guide private capital to actively participate in green development.

Translation B. We will set up the State Green Transition Capital Fund to support the transition of traditional energy industries toward clean and low-carbon development, and guide private capital to actively participate in green development.

Translation C. We will set up the China's Carbon Transition Finance Facility to support the transition of traditional energy industries toward clean and low-carbon development, and guide private capital to actively participate in green development.

Translation D. We will set up the National Clean Energy Transition Investment Fund to support the transition of traditional energy industries toward clean and low-carbon development, and guide private capital to actively participate in green development.

Translation E. We will set up the National Low-Carbon Transition Fund (guójiā dītàn zhuǎnxíng jījīn) to support the transition of traditional energy industries toward clean and low-carbon development, and guide private capital to actively participate in green development.

[N-05] Term: "内卷式"竞争

Translation A. We will strengthen anti-monopoly and anti-unfair competition efforts, make integrated use of capacity regulation, standard-setting and price law enforcement, and intensify efforts to address "involution-type" competition, so as to foster a sound market ecosystem.

Translation B. We will strengthen anti-monopoly and anti-unfair competition efforts, make integrated use of capacity regulation, standard-setting and price law enforcement, and intensify efforts to address rat-race competition, so as to foster a sound market ecosystem.

Translation C. We will strengthen anti-monopoly and anti-unfair competition efforts, make integrated use of capacity regulation, standard-setting and price law enforcement, and intensify efforts to address destructive price undercutting, so as to foster a sound market ecosystem.

Translation D. We will strengthen anti-monopoly and anti-unfair competition efforts, make integrated use of capacity regulation, standard-setting and price law enforcement, and intensify efforts to address excessive and inefficient market competition, so as to foster a sound market ecosystem.

Translation E. We will strengthen anti-monopoly and anti-unfair competition efforts, make integrated use of capacity regulation, standard-setting and price law enforcement, and intensify efforts to address nèijuǎn-style competition (involution-driven market behavior), so as to foster a sound market ecosystem.

[N-06] Term: 碳排放双控

Translation A. We will steadily advance the development of the dual control of carbon emissions system, incorporate the reduction of carbon dioxide emissions per unit of GDP into annual binding target management, and refine supporting policies and measures.

Translation B. We will steadily advance the development of the carbon emissions twin-track control system, incorporate the reduction of carbon dioxide emissions per unit of GDP into annual binding target management, and refine supporting policies and measures.

Translation C. We will steadily advance the development of the carbon intensity double-constraint mechanism system, incorporate the reduction of carbon dioxide emissions per unit of GDP into annual binding target management, and refine supporting policies and measures.

Translation D. We will steadily advance the development of the integrated carbon emission reduction management system, incorporate the reduction of carbon dioxide emissions per unit of GDP into annual binding target management, and refine supporting policies and measures.

Translation E. We will steadily advance the development of the dual-control over carbon emissions (shuāngkòng) system, incorporate the reduction of carbon dioxide emissions per unit of GDP into annual binding target management, and refine supporting policies and measures.

[N-07] Term: 智能原生

Translation A. We will deepen and expand the AI Plus Initiative, foster new forms and models of intelligence-native business, and support the thriving development of the open-source ecosystem.

Translation B. We will deepen and expand the AI Plus Initiative, foster new forms and models of AI-native business, and support the thriving development of the open-source ecosystem.

Translation C. We will deepen and expand the AI Plus Initiative, foster new forms and models of AI-first native applications business, and support the thriving development of the open-source ecosystem.

Translation D. We will deepen and expand the AI Plus Initiative, foster new forms and models of AI-integrated native applications business, and support the thriving development of the open-source ecosystem.

Translation E. We will deepen and expand the AI Plus Initiative, foster new forms and models of intelligent-native (zhìnéng yuánshēng) business, and support the thriving development of the open-source ecosystem.

[N-08] Term: 未来能源

Translation A. We will make forward-looking plans for strategic frontiers including future energy, quantum technology, and brain-computer interfaces, and establish mechanisms for investment growth and risk-sharing in future industries.

Translation B. We will make forward-looking plans for strategic frontiers including next-generation energy, quantum technology, and brain-computer interfaces, and establish mechanisms for investment growth and risk-sharing in future industries.

Translation C. We will make forward-looking plans for strategic frontiers including advanced energy systems, quantum technology, and brain-computer interfaces, and establish mechanisms for investment growth and risk-sharing in future industries.

Translation D. We will make forward-looking plans for strategic frontiers including emerging and innovative energy sources, quantum technology, and brain-computer interfaces, and establish mechanisms for investment growth and risk-sharing in future industries.

Translation E. We will make forward-looking plans for strategic frontiers including wèilái energy (frontier energy systems), quantum technology, and brain-computer interfaces, and establish mechanisms for investment growth and risk-sharing in future industries.

[N-09] Term: 养老服务消费补贴

Translation A. We will pilot elderly care service consumption subsidies schemes, support residents in purchasing diversified elderly care services including home-visit care and community day-care, and expand elderly care consumption.

Translation B. We will pilot senior care service vouchers schemes, support residents in purchasing diversified elderly care services including home-visit care and community day-care, and expand elderly care consumption.

Translation C. We will pilot care voucher scheme for older adults schemes, support residents in purchasing diversified elderly care services including home-visit care and community day-care, and expand elderly care consumption.

Translation D. We will pilot elderly care spending support program schemes, support residents in purchasing diversified elderly care services including home-visit care and community day-care, and expand elderly care consumption.

Translation E. We will pilot elderly care service consumption subsidies (yǎnglǎo fúwù xiāofèi bǔtiē) schemes, support residents in purchasing diversified elderly care services including home-visit care and community day-care, and expand elderly care consumption.

[N-10] Term: 扩能提质

Translation A. We will expand capacity and improve quality in the service sector, with a focus on supporting consumer services in addressing shortcomings and improving quality, and expanding service supply in commerce and distribution, culture and tourism, and healthcare.

Translation B. We will scale up and upgrade the service sector, with a focus on supporting consumer services in addressing shortcomings and improving quality, and expanding service supply in commerce and distribution, culture and tourism, and healthcare.

Translation C. We will broaden and upgrade the service sector, with a focus on supporting consumer services in addressing shortcomings and improving quality, and expanding service supply in commerce and distribution, culture and tourism, and healthcare.

Translation D. We will develop and enhance the quality of the service sector, with a focus on supporting consumer services in addressing shortcomings and improving quality, and expanding service supply in commerce and distribution, culture and tourism, and healthcare.

Translation E. We will kuò néng tí zhì (expand capacity while raising quality standards in) the service sector, with a focus on supporting consumer services in addressing shortcomings and improving quality, and expanding service supply in commerce and distribution, culture and tourism, and healthcare.

============================================================

Please provide rankings for all 20 terms in the required format:

# 3 Detailed Table of Model Ranking Stability at the Lexical Level

Appendix C presents the Kendall's W coefficient of concordance and Friedman test results for all 8 models across the 20 stimulus terms, serving as the complete data source for the stability analysis in Section 5.2 of the main text. The criteria for stability are: Kendall's W ≥ 0.70 and Friedman p < .10 (a relaxed threshold is adopted considering the small sample size of 5 ranking items). Those meeting both criteria are marked with '✓', otherwise '✗'. Term IDs starting with 'O-' denote classic political discourse, while those starting with 'N-' denote emerging tech & policy discourse.

| ID | Source Term | Kendall's W | Friedman p | Stability |
| --- | --- | --- | --- | --- |
| O-01 | hiding one's capabilities while biding one's time | 0.822 | .043 | ✓ |
| O-02 | a community with a shared future for mankind | 0.889 | .031 | ✓ |
| O-03 | governing the country in accordance with law | 0.711 | .074 | ✓ |
| O-04 | the Belt and Road | 0.956 | .022 | ✓ |
| O-05 | new quality productive forces | 0.911 | .027 | ✓ |
| O-06 | propaganda | 0.911 | .027 | ✓ |
| O-07 | united front | 0.844 | .038 | ✓ |
| O-08 | safeguard rights and interests | 0.867 | .034 | ✓ |
| O-09 | socialism with Chinese characteristics | 0.956 | .022 | ✓ |
| O-10 | ecological civilization | 0.889 | .031 | ✓ |
| N-01 | new form of intelligent economy | 0.822 | .043 | ✓ |
| N-02 | embodied intelligence | 0.733 | .066 | ✓ |
| N-03 | computing-power and electricity coordination | 0.911 | .027 | ✓ |
| N-04 | National Low-Carbon Transition Fund | 0.956 | .022 | ✓ |
| N-05 | "involution-type" competition | 0.600 | .126 | ✗ |
| N-06 | dual control of carbon emissions | 0.489 | .209 | ✗ |
| N-07 | intelligence-native | 0.778 | .053 | ✓ |
| N-08 | future energy | 0.644 | .102 | ✗ |
| N-09 | elderly care service consumption subsidies | 0.578 | .140 | ✗ |
| N-10 | expand capacity and improve quality | 0.956 | .022 | ✓ |

Table C-1: Ranking Stability of DeepSeek-V3.2 (CN) across Terms

| ID | Source Term | Kendall's W | Friedman p | Stability |
| --- | --- | --- | --- | --- |
| O-01 | hiding one's capabilities while biding one's time | 0.911 | .027 | ✓ |
| O-02 | a community with a shared future for mankind | 0.844 | .038 | ✓ |
| O-03 | governing the country in accordance with law | 0.889 | .031 | ✓ |
| O-04 | the Belt and Road | 0.822 | .043 | ✓ |
| O-05 | new quality productive forces | 0.822 | .043 | ✓ |
| O-06 | propaganda | 0.867 | .034 | ✓ |
| O-07 | united front | 0.822 | .043 | ✓ |
| O-08 | safeguard rights and interests | 0.889 | .031 | ✓ |
| O-09 | socialism with Chinese characteristics | 0.956 | .022 | ✓ |
| O-10 | ecological civilization | 1.000 | .017 | ✓ |
| N-01 | new form of intelligent economy | 1.000 | .017 | ✓ |
| N-02 | embodied intelligence | 0.956 | .022 | ✓ |
| N-03 | computing-power and electricity coordination | 0.822 | .043 | ✓ |
| N-04 | National Low-Carbon Transition Fund | 0.889 | .031 | ✓ |
| N-05 | "involution-type" competition | 0.822 | .043 | ✓ |
| N-06 | dual control of carbon emissions | 0.956 | .022 | ✓ |
| N-07 | intelligence-native | 1.000 | .017 | ✓ |
| N-08 | future energy | 1.000 | .017 | ✓ |
| N-09 | elderly care service consumption subsidies | 1.000 | .017 | ✓ |
| N-10 | expand capacity and improve quality | 0.956 | .022 | ✓ |

Table C-2: Ranking Stability of Gemini-3-Pro (US) across Terms

| ID | Source Term | Kendall's W | Friedman p | Stability |
| --- | --- | --- | --- | --- |
| O-01 | hiding one's capabilities while biding one's time | 0.667 | .092 | ✗ |
| O-02 | a community with a shared future for mankind | 0.956 | .022 | ✓ |
| O-03 | governing the country in accordance with law | 0.689 | .082 | ✗ |
| O-04 | the Belt and Road | 0.822 | .043 | ✓ |
| O-05 | new quality productive forces | 0.911 | .027 | ✓ |
| O-06 | propaganda | 0.844 | .038 | ✓ |
| O-07 | united front | 0.600 | .126 | ✗ |
| O-08 | safeguard rights and interests | 0.778 | .053 | ✗ |
| O-09 | socialism with Chinese characteristics | 0.867 | .034 | ✓ |
| O-10 | ecological civilization | 0.956 | .022 | ✓ |
| N-01 | new form of intelligent economy | 0.911 | .027 | ✓ |
| N-02 | embodied intelligence | 0.911 | .027 | ✓ |
| N-03 | computing-power and electricity coordination | 0.800 | .048 | ✓ |
| N-04 | National Low-Carbon Transition Fund | 0.556 | .154 | ✗ |
| N-05 | "involution-type" competition | 0.911 | .027 | ✓ |
| N-06 | dual control of carbon emissions | 0.733 | .066 | ✗ |
| N-07 | intelligence-native | 0.911 | .027 | ✓ |
| N-08 | future energy | 1.000 | .017 | ✓ |
| N-09 | elderly care service consumption subsidies | 0.600 | .126 | ✗ |
| N-10 | expand capacity and improve quality | 1.000 | .017 | ✓ |

Table C-3: Ranking Stability of Kimi-2.5 (CN) across Terms

| ID | Source Term | Kendall's W | Friedman p | Stability |
| --- | --- | --- | --- | --- |
| O-01 | hiding one's capabilities while biding one's time | 0.844 | .038 | ✓ |
| O-02 | a community with a shared future for mankind | 0.956 | .022 | ✓ |
| O-03 | governing the country in accordance with law | 0.889 | .031 | ✓ |
| O-04 | the Belt and Road | 1.000 | .017 | ✓ |
| O-05 | new quality productive forces | 0.733 | .066 | ✗ |
| O-06 | propaganda | 0.733 | .066 | ✗ |
| O-07 | united front | 0.689 | .082 | ✗ |
| O-08 | safeguard rights and interests | 0.889 | .031 | ✓ |
| O-09 | socialism with Chinese characteristics | 0.956 | .022 | ✓ |
| O-10 | ecological civilization | 0.956 | .022 | ✓ |
| N-01 | new form of intelligent economy | 0.956 | .022 | ✓ |
| N-02 | embodied intelligence | 0.956 | .022 | ✓ |
| N-03 | computing-power and electricity coordination | 0.889 | .031 | ✓ |
| N-04 | National Low-Carbon Transition Fund | 0.689 | .082 | ✗ |
| N-05 | "involution-type" competition | 0.956 | .022 | ✓ |
| N-06 | dual control of carbon emissions | 0.556 | .172 | ✗ |
| N-07 | intelligence-native | 0.889 | .031 | ✓ |
| N-08 | future energy | 0.956 | .022 | ✓ |
| N-09 | elderly care service consumption subsidies | 0.622 | .126 | ✗ |
| N-10 | expand capacity and improve quality | 0.956 | .022 | ✓ |

Table C-4: Ranking Stability of GPT-5.3 (US) across Terms

| ID | Source Term | Kendall's W | Friedman p | Stability |
| --- | --- | --- | --- | --- |
| O-01 | hiding one's capabilities while biding one's time | 1.000 | .017 | ✓ |
| O-02 | a community with a shared future for mankind | 0.956 | .022 | ✓ |
| O-03 | governing the country in accordance with law | 0.956 | .022 | ✓ |
| O-04 | the Belt and Road | 1.000 | .017 | ✓ |
| O-05 | new quality productive forces | 0.822 | .043 | ✓ |
| O-06 | propaganda | 0.956 | .022 | ✓ |
| O-07 | united front | 0.867 | .034 | ✓ |
| O-08 | safeguard rights and interests | 0.867 | .034 | ✓ |
| O-09 | socialism with Chinese characteristics | 1.000 | .017 | ✓ |
| O-10 | ecological civilization | 0.956 | .022 | ✓ |
| N-01 | new form of intelligent economy | 0.956 | .022 | ✓ |
| N-02 | embodied intelligence | 0.867 | .034 | ✓ |
| N-03 | computing-power and electricity coordination | 0.622 | .113 | ✗ |
| N-04 | National Low-Carbon Transition Fund | 0.956 | .022 | ✓ |
| N-05 | "involution-type" competition | 1.000 | .017 | ✓ |
| N-06 | dual control of carbon emissions | 0.889 | .031 | ✓ |
| N-07 | intelligence-native | 0.733 | .066 | ✗ |
| N-08 | future energy | 0.911 | .025 | ✓ |
| N-09 | elderly care service consumption subsidies | 1.000 | .017 | ✓ |
| N-10 | expand capacity and improve quality | 0.956 | .022 | ✓ |

Table C-5: Ranking Stability of Grok 4.2 (US) across Terms

| ID | Source Term | Kendall's W | Friedman p | Stability |
| --- | --- | --- | --- | --- |
| O-01 | hiding one's capabilities while biding one's time | 0.489 | .209 | ✗ |
| O-02 | a community with a shared future for mankind | 0.711 | .074 | ✗ |
| O-03 | governing the country in accordance with law | 0.911 | .027 | ✓ |
| O-04 | the Belt and Road | 0.911 | .027 | ✓ |
| O-05 | new quality productive forces | 0.356 | .370 | ✗ |
| O-06 | propaganda | 0.956 | .022 | ✓ |
| O-07 | united front | 0.800 | .048 | ✓ |
| O-08 | safeguard rights and interests | 0.822 | .043 | ✓ |
| O-09 | socialism with Chinese characteristics | 0.822 | .043 | ✓ |
| O-10 | ecological civilization | 0.867 | .034 | ✓ |
| N-01 | new form of intelligent economy | 0.822 | .043 | ✓ |
| N-02 | embodied intelligence | 0.733 | .066 | ✗ |
| N-03 | computing-power and electricity coordination | 0.622 | .113 | ✗ |
| N-04 | National Low-Carbon Transition Fund | 0.867 | .034 | ✓ |
| N-05 | "involution-type" competition | 0.733 | .066 | ✗ |
| N-06 | dual control of carbon emissions | 0.689 | .082 | ✗ |
| N-07 | intelligence-native | 0.867 | .034 | ✓ |
| N-08 | future energy | 0.622 | .113 | ✗ |
| N-09 | elderly care service consumption subsidies | 0.489 | .209 | ✗ |
| N-10 | expand capacity and improve quality | 0.911 | .027 | ✓ |

Table C-6: Ranking Stability of Qwen-3.5 (CN) across Terms

| ID | Source Term | Kendall's W | Friedman p | Stability |
| --- | --- | --- | --- | --- |
| O-01 | hiding one's capabilities while biding one's time | 0.311 | .448 | ✗ |
| O-02 | a community with a shared future for mankind | 0.956 | .022 | ✓ |
| O-03 | governing the country in accordance with law | 0.867 | .034 | ✓ |
| O-04 | the Belt and Road | 0.956 | .022 | ✓ |
| O-05 | new quality productive forces | 0.911 | .027 | ✓ |
| O-06 | propaganda | 0.956 | .022 | ✓ |
| O-07 | united front | 0.956 | .022 | ✓ |
| O-08 | safeguard rights and interests | 0.911 | .027 | ✓ |
| O-09 | socialism with Chinese characteristics | 1.000 | .017 | ✓ |
| O-10 | ecological civilization | 0.956 | .022 | ✓ |
| N-01 | new form of intelligent economy | 0.956 | .022 | ✓ |
| N-02 | embodied intelligence | 0.867 | .034 | ✓ |
| N-03 | computing-power and electricity coordination | 0.911 | .027 | ✓ |
| N-04 | National Low-Carbon Transition Fund | 0.956 | .022 | ✓ |
| N-05 | "involution-type" competition | 0.956 | .022 | ✓ |
| N-06 | dual control of carbon emissions | 0.867 | .034 | ✓ |
| N-07 | intelligence-native | 0.867 | .034 | ✓ |
| N-08 | future energy | 1.000 | .017 | ✓ |
| N-09 | elderly care service consumption subsidies | 0.822 | .043 | ✓ |
| N-10 | expand capacity and improve quality | 0.733 | .066 | ✗ |

Table C-7: Ranking Stability of Claude Sonnet 4.6 (US) across Terms

| ID | Source Term | Kendall's W | Friedman p | Stability |
| --- | --- | --- | --- | --- |
| O-01 | hiding one's capabilities while biding one's time | 0.822 | .043 | ✓ |
| O-02 | a community with a shared future for mankind | 0.889 | .031 | ✓ |
| O-03 | governing the country in accordance with law | 0.889 | .031 | ✓ |
| O-04 | the Belt and Road | 0.956 | .022 | ✓ |
| O-05 | new quality productive forces | 0.822 | .043 | ✓ |
| O-06 | propaganda | 0.956 | .022 | ✓ |
| O-07 | united front | 0.889 | .031 | ✓ |
| O-08 | safeguard rights and interests | 0.467 | >.05 | ✗ |
| O-09 | socialism with Chinese characteristics | 1.000 | .017 | ✓ |
| O-10 | ecological civilization | 0.956 | .022 | ✓ |
| N-01 | new form of intelligent economy | 0.956 | .022 | ✓ |
| N-02 | embodied intelligence | 0.956 | .022 | ✓ |
| N-03 | computing-power and electricity coordination | 0.956 | .022 | ✓ |
| N-04 | National Low-Carbon Transition Fund | 0.867 | .034 | ✓ |
| N-05 | "involution-type" competition | 0.800 | .048 | ✓ |
| N-06 | dual control of carbon emissions | 0.889 | .031 | ✓ |
| N-07 | intelligence-native | 0.911 | .027 | ✓ |
| N-08 | future energy | 0.822 | .043 | ✓ |
| N-09 | elderly care service consumption subsidies | 0.956 | .022 | ✓ |
| N-10 | expand capacity and improve quality | 0.822 | .043 | ✓ |

Table C-8: Ranking Stability of ERNIE 5.0 (CN) across Terms

| Source Term | Category | Frequency of Instability (out of 8 models) |
| --- | --- | --- |
| elderly care service consumption subsidies | Emerging | 4 |
| dual control of carbon emissions | Emerging | 4 |
| hiding one's capabilities while biding one's time | Classic | 3 |
| "involution-type" competition | Emerging | 2 |
| new quality productive forces | Emerging | 2 |
| future energy | Emerging | 2 |
| National Low-Carbon Transition Fund | Emerging | 2 |
| united front | Classic | 2 |
| safeguard rights and interests | Classic | 2 |
| computing-power and electricity coordination | Emerging | 2 |
| expand capacity and improve quality | Emerging | 1 |
| embodied intelligence | Emerging | 1 |
| a community with a shared future for mankind | Classic | 1 |
| propaganda | Classic | 1 |
| governing the country in accordance with law | Classic | 1 |
| intelligence-native | Emerging | 1 |

Table C-9: Cross-Model Frequency Distribution of Unstable Terms

Appendix D: Complete Statistical Results of Between-Group Differences Tests

Appendix D presents the full set of statistics for the Mann-Whitney U test and Welch's t-test reported in Section 5.3 of the main text, providing BH-FDR multiple comparison correction results and the cross-group means by term category, ensuring reproducibility and transparency.

| TNO Dimension | CN M (SD) | US M (SD) | CN Median | US Median | U | p (MWU) | q (BH-FDR) | Cliff's δ | Welch's t | p (Welch) |
| --- | --- | --- | --- | --- | --- | --- | --- | --- | --- | --- |
| FN | 1.567 (0.324) | 1.268 (0.099) | 1.498 | 1.291 | 12 | .343 | 1.000 | +0.500 | 1.524 | .211 |
| FLN | 2.667 (0.165) | 2.588 (0.341) | 2.613 | 2.520 | 9 | .886 | 1.000 | +0.125 | 0.362 | .735 |
| CAN | 3.567 (0.354) | 3.644 (0.180) | 3.505 | 3.633 | 6 | .686 | 1.000 | −0.250 | −0.332 | .755 |
| EHN | 3.608 (0.268) | 3.618 (0.394) | 3.566 | 3.571 | 8 | 1.000 | 1.000 | 0.000 | −0.036 | .973 |
| FRN | 3.589 (0.514) | 3.882 (0.806) | 3.694 | 4.078 | 7 | .886 | 1.000 | −0.125 | −0.530 | .618 |

Table D-1: Between-Group Differences across Five TNO Dimensions: Mann-Whitney U Test and Welch's t-test

Note 1: CN n = 4 (DeepSeek-V3.2, Kimi-2.5, Qwen-3.5, ERNIE 5.0), US n = 4 (Claude Sonnet 4.6, GPT-5.3, Gemini-3-Pro, Grok 4.2).

Note 2: BH-FDR correction is based on the sequence of original p-values from 5 independent comparisons. All corrected q-values are 1.000, reflecting that the original p-values are far above any reasonable significance threshold, rather than indicating a calculation anomaly in the correction procedure.

Note 3: A positive Cliff's δ indicates a tendency for the CN group to score higher than the US group, while a negative value indicates the opposite. For the FN dimension, Cliff's δ = +0.500, reaching the threshold for a medium effect size as defined by Cohen (1988) (|δ| ≥ 0.474). This is marked as an exploratory finding and should not be interpreted as confirmatory.

The following table further breaks down the four-way crossed means by term category (classic political discourse/emerging tech & policy discourse) and model group (CN/US), providing the complete data basis for the discourse moderation effect analysis in Section 5.1.2.

| Group | Term Category | FN | FLN | CAN | EHN | FRN |
| --- | --- | --- | --- | --- | --- | --- |
| CN | Classic Political Discourse | 1.656 | 2.853 | 3.708 | 3.500 | 3.281 |
| CN | Emerging Tech & Policy Discourse | 1.431 | 2.518 | 3.493 | 3.802 | 3.753 |
| US | Classic Political Discourse | 1.342 | 2.749 | 3.703 | 3.537 | 3.667 |
| US | Emerging Tech & Policy Discourse | 1.176 | 2.441 | 3.569 | 3.745 | 4.069 |

Table D-2: TNO Means Crossed by Term Category and Group

Note: The discourse moderation effect for the EHN dimension is consistent in direction across both groups (emerging discourse is higher than classic discourse). The difference is +0.302 for the CN group and +0.208 for the US group, indicating that the activating effect of emerging tech & policy discourse on ethical hedging tendencies transcends national boundaries and holds a certain universality. The systematic increase of the FRN dimension in emerging discourse (CN: +0.472; US: +0.402) suggests that while emerging discourse triggers stronger ethical hedging tendencies, it also produces a parallel repulsive effect on foreignization. The simultaneous shift in both dimensions may point to a 'hedged faithfulness' normative response pattern: neither foreignizing nor actively softening, but retreating to the safe zone of semantic equivalence.
